# Synthesis and In Vitro Anticancer Evaluation of Novel Phosphonium Derivatives of Chrysin

**DOI:** 10.3390/ijms262211063

**Published:** 2025-11-15

**Authors:** Mónika Halmai, Dominika Mária Herr, Szabolcs Mayer, Péter Keglevich, Ejlal A. Abdallah, Noémi Bózsity-Faragó, István Zupkó, Andrea Nehr-Majoros, Éva Szőke, Zsuzsanna Helyes, László Hazai

**Affiliations:** 1 Department of Organic Chemistry and Technology, Faculty of Chemical Technology and Biotechnology, Budapest University of Technology and Economics, Műegyetem rkp. 3, H-1111 Budapest, Hungary; monika.halmai@edu.bme.hu (M.H.);; 2 Institute of Pharmacodynamics and Biopharmacy, University of Szeged, Eötvös u. 6, H-6720 Szeged, Hungary; 3 Department of Pharmacology and Pharmacotherapy, Medical School & Centre for Neuroscience, University of Pécs, Szigeti út 12, H-7624 Pécs, Hungary; 4 National Laboratory for Drug Research and Development, Magyar Tudósok krt. 2, H-1117 Budapest, Hungary; 5 HUN-REN PTE Chronic Pain Research Group, Szigeti út 12, H-7624 Pécs, Hungary; 6 PharmInVivo Ltd., Szondi Gy. u. 10, H-7629 Pécs, Hungary

**Keywords:** chrysin, trisubstituted phosphines, hybrids, phosphonium salts, mitochondrial accumulation, antiproliferative effect, cell viability

## Abstract

One of the best-known flavonoid chrysin was coupled at position 7 with several trisubstituted phosphine derivatives with a flexible spacer, and their in vitro anticancer activities were investigated on 60 human tumor cell lines (NCI60) and on several gynecological cancer cells. The trisubstituted phosphines contained different substituents on the aromatic ring(s), e.g., methyl and methoxy groups or fluoro atoms. The phosphorus atom was substituted not only with aromatic rings but with cyclohexyl substituents. The ionic phosphonium building block is important because it allows the therapeutic agents to transfer across the cell membrane. Therefore, the pharmacophores linked to it can exert their effects in the mitochondria. Instead of the ionic phosphonium element, a neutral moiety, namely the triphenylmethyl group, was also added to the side chain, being sterically similar but without a charge and phosphorus atom. Most of the hybrids exhibited low micromolar growth inhibition (*GI*_50_) values against the majority of the tested cell lines. Notably, conjugate **3f** stood out, demonstrating nanomolar antitumor activity against the K-562 leukemia cell line (*GI*_50_ = 34 nM). One selected compound (**3i**) with promising cancer selectivity elicited cell cycle disturbances and inhibited the migration of breast cancer. The tumor-selectivity of **3a** and **3f** was assessed based on their effects on non-tumor Chinese hamster ovary (CHO) cells using the CellTiter-Glo Luminescent Cell Viability Assay. Given their estimated half-maximal inhibitory concentration (*IC*_50_) values on non-tumor CHO cells (2.65 µM and 1.15 µM, respectively), these conjugates demonstrate promising selectivity toward several cancer cell lines. The excellent results obtained may serve as good starting points for further optimization and the design of even more effective flavonoid- and/or phosphonium-based drugs.

## 1. Introduction

**Figure 1:**
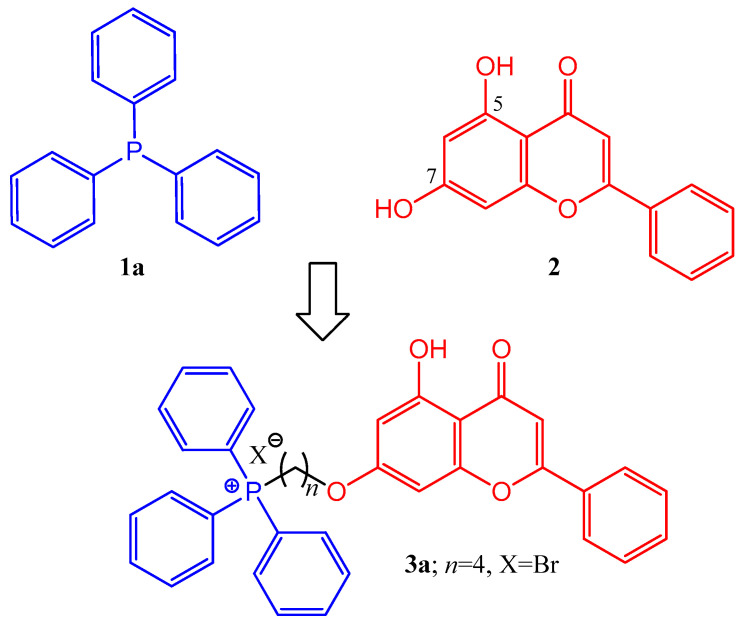
Triphenylphosphine (**1a**), chrysin (**2**), and one of the chrysin–TPP conjugates (**3a**) planned for synthesis.

The best-known representative of trisubstituted phosphines is triphenylphosphine (TPP) (**1a**) (Figure 1), which has recently played an increasingly important role in the synthesis of anticancer hybrids [1]. An opportunity to target tumor cells is to deliver medicines into mitochondria. Four main approaches have been elaborated: applying lipophilic cations, the application of cell-penetrating peptides, the use of nanoparticles, and physical penetration. The most frequently used method is the transfer of lipophilic cations [2]. Triphenylphosphonium (TPP^+^)-based cations have a hydrophobic surface and can readily diffuse across the inner membrane. Moreover, TPP^+^ cations have been demonstrated to localize to the mitochondrial matrix at concentrations 1000-fold higher than those in the cytoplasm [3]. Since tumor cells have increased mitochondrial and plasma membrane potential compared to non-cancerous cells, the TPP+ cation may be able to induce selective cell death [4]. Nevertheless, TPP derivatives may have the advantages that TPP is relatively stable in biological systems, is both hydrophilic and lipophilic, is relatively easy to synthesize and purify, has low chemical reactivity with cellular components, and has no light absorption or fluorescence emission in the visible and infrared spectral regions [5]. In addition, TPP is more likely to transport drug molecules across the membrane barrier and is safer than other lipophilic cations [1]. In addition, it is expected that the tolyl and methoxy groups on the phenyl ring(s) stabilize the cation due to their electron-donating nature, which may enhance its ability to diffuse into the mitochondrial matrix, which in turn may lead to accumulation, increasing efficiency [3].

Based on these biological concepts, triterpene–TPP hybrids were synthesized, and it was found that the anticancer activity can be significantly increased by the synthesis of new molecular hybrids of betulin via covalent linkage with, e.g., an alkyltriphenylphosphonium moiety [4,6,7].

Recently, a paper was published by us in which derivatives of the *Vinca* alkaloid vindoline with trisubstituted phosphines were presented [8]. In the course of the biological investigations of the compounds, it was found that some of the derivatives showed significant antiproliferative activity with *GI*_50_ values in the nanomolar range.

Flavonoids are plant metabolites with a polyphenolic structure that occur in many places in nature (fruits, vegetables, flowers, etc.). These compounds have a wide spectrum of biological activities, including antibacterial, anti-inflammatory, antioxidant, and anticancer effects [9]. Flavonoids have also been conjugated with TPP through linkers [10]. Several quercetin derivatives showed significant cytostatic/cytotoxic effects, and it was found that the compounds accumulate in the mitochondria by a transmembrane potential-driven procedure [11,12,13,14]. Other polyphenols were also investigated; thus, TPP derivatives of the antioxidant resveratrol, which is also presumed to have cancer-preventing effects, were also synthesized. The accumulation of hybrids in rat liver mitochondria is described using various biological methods [15,16].

One of the best-known representatives of flavones is chrysin (5,7-dihydroxyflavone) (**2**) (Figure 1), which is found in blue passionflower (*Passiflora caerulea*), honey, and propolis [17]. Studies have shown that chrysin (**2**) has cytotoxic effects by stimulating apoptosis in cells [18,19,20]. It also plays an important role in inhibiting tumor growth and neoplasticity [21,22]. Due to these beneficial properties, it may also be suitable for the production of antiproliferative derivatives and hybrid molecules [23,24,25,26,27,28,29,30,31,32,33,34].

**Figure 2:**
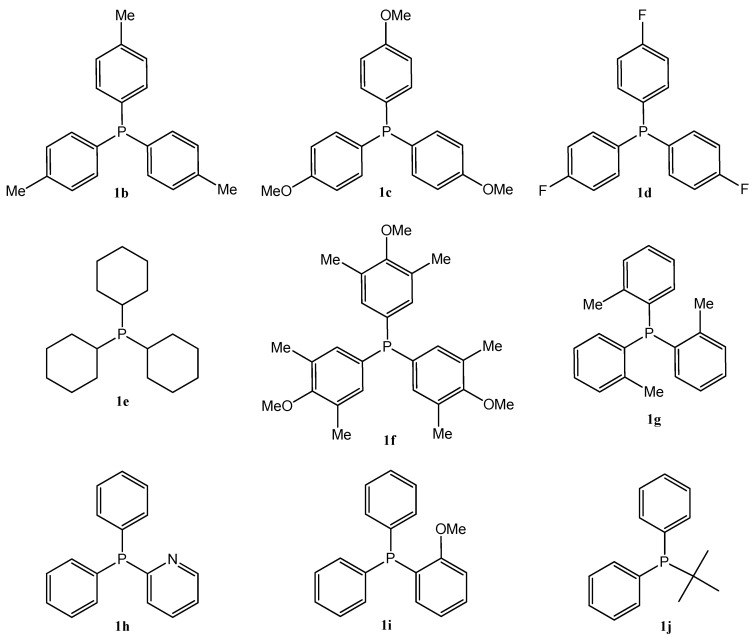
The structure of the selected trisubstituted phosphines (**1b**–**1j**).

Based on the summarized biological principles, our goal was the synthesis of chrysin hybrids, in which chrysin was coupled with trisubstituted phosphine derivatives, and the investigation of the antitumor activity of the obtained compounds. To the best of our knowledge, this is the first report on the synthesis of a chrysin-based hybrid incorporating a pharmacophore that presumably exerts its effect in the mitochondria. A 4-carbon linker was used for alkylation of the hydroxy group at position 7 of chrysin and the phosphorus atom of the trisubstituted phosphin derivatives. The simplest derivative **3a** is presented in Figure 1. The chosen trisubstituted phosphins are shown in Figure 2. We used symmetrically substituted phosphines with methyl and methoxy groups (**1b**,**c**,**f**,**g**), with fluoro atoms (**1d**), and also the tricyclohexyl derivative **1e**, investigating the role of the aromatic character. Further phosphine derivatives were a pyridyl derivative (**1h**), a monomethoxy substituted (**1i**), and a diphenylalkyl (*tert*-butyl) substituted phosphine (**1j**). In this way, the effect of the heterocyclic and aliphatic substituents on the trisubstituted phosphines was also investigated with these simple models.

## 2. Results and Discussion

### 2.1. Chemistry

**Scheme 1:**
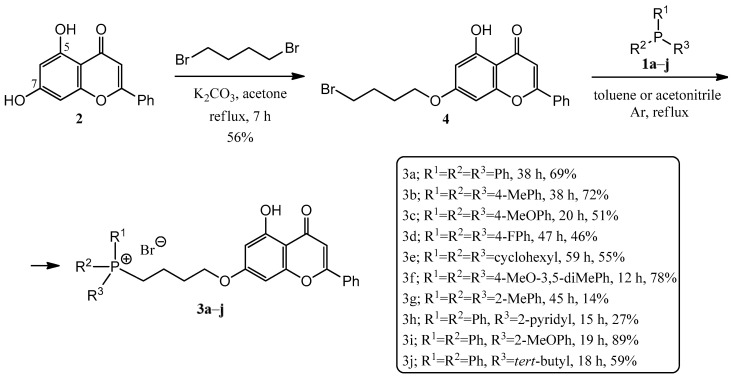
Synthesis of chrysin–TPP hybrids (**3a**–**j**).

In order to build the phosphine derivatives into position 7 of chrysin (**2**), the formation of a linker was necessary. When choosing the type and the length of the linker, we took into account the results obtained in the course of the previous syntheses of similar hybrids [8,10,11,24,35]. Based on this, a 4-carbon linker was selected. Thus, chrysin (**2**) was mono-*O*-alkylated with 1,4-dibromobutane in the presence of potassium carbonate as a base in refluxing acetone, utilizing the method of Babu et al. [35] (Scheme 1). The reason for the regioselectivity is that the other phenolic hydroxyl group at position 5 of chrysin (**2**) forms an intramolecular hydrogen bond with the neighboring oxo group.

Then, the alkylated chrysin derivative (**4**) was reacted with corresponding phosphines (**1a**–**j**) in toluene (**a**–**e**,**h**) or acetonitrile (**f**–**g**,**i**–**j**) solvent (Scheme 1). The solution was refluxed under Ar atmosphere until the starting materials were consumed (for 12–59 h). The crude products were purified by preparative TLC, obtaining the expected phosphonium salts (**3a**–**j**). The yields were in good agreement with expectations, as the *P*-alkylations were carried out in good yields with phosphines containing electron-donating groups (e.g., **1f**, 78%, the *P* atom is more nucleophilic), while phosphines substituted with electron-withdrawing groups (e.g., **1d**, 46%, the *P* atom is less nucleophilic) or substituted in the ortho position (**1g**, 14% steric hindrance) resulted in poorer yields.

In the course of the research, it was planned to prove that the positively charged phosphorus atom significantly influences the antitumor effect of hybrid molecules. For this purpose, a neutral element was connected to chrysin (**2**) at position 7, namely a triphenylmethyl group, which is sterically similar but does not contain an ionic charge.

**Scheme 2:**
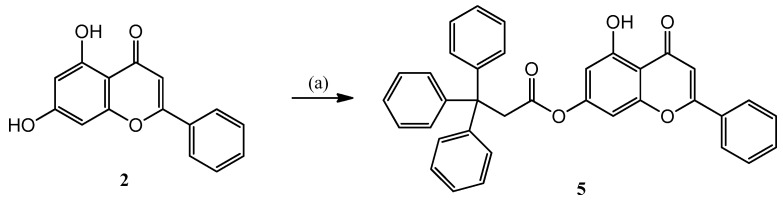
Synthesis of **5** derivative containing triphenylmethyl group at position 7 of chrysin (**2**): (a) 3,3,3-triphenylpropionic acid, SOCl_2_, abs. DCM, DMF, 60 °C, 5 h; then chrysin (**2**), NEt_3_, acetone, 45 °C, 6 h, 60%.

Therefore, chrysin (**2**) was *O*-acylated with 3,3,3-triphenylpropanoyl chloride prepared in situ from the corresponding carboxylic acid in acetone solution in the presence of triethyl amine, resulting in the **5** ester (Scheme 2).

### 2.2. Biological Evaluation

#### 2.2.1. NCI60 Screening

The in vitro antiproliferative effects of the prepared phosphonium salts (**3a**–**h**) were studied on 60 human tumor cell lines (NCI60), representing leukemia, non-small-cell lung, colon, CNS, ovarian, renal, prostate, and breast cancers, as well as melanoma, at the National Cancer Institute (NCI, USA) according to the standard protocols [36,37,38,39,40,41].

The screening results are presented in the Supplementary Materials (Table S1), where the cytotoxicity was determined for the 10 µM concentration. The percentages of growths demonstrate the amount of living cancer cells compared to a reference. The negative numbers indicate significant decreases in the cell number. In our previous work, we have shown that chrysin (**2**) does not exert remarkable antiproliferative effects [24,26]. Compared to the reference chrysin (**2**), all phosphonium hybrids (**3a**–**h**) had significant antiproliferative activities. Among them, compound **3g** demonstrated the most potent and broad-spectrum cytotoxic activity, with a mean inhibition of −97.65%, exhibiting near-complete growth suppression in several cell lines, including leukemia non-small-cell lung cancer (NCI-H226: −99.39%), melanoma (UACC-257: −99.31%), ovarian cancer (IGROV1: −99.12%), renal cancer (TK-10: −99.33%) and breast cancer (MDA-MB-468: −99.41%). At the same time, it should be noted that more than 60% average reduction was also obtained in the case of the other examined conjugates (**3a**–**h**).

Since all new conjugates (**3a**–**h**) had notable antiproliferative activities on several tumor cell lines in the course of the one-dose screening, they were subjected to a five-dose test. The 50% growth inhibition (*GI*_50_) and their mean values are presented in Table 1. The results revealed that compound **3f** exhibited the most potent overall activity, with a mean *GI*_50_ of 0.312 µM, followed closely by compound **3b** (0.322 µM), **3g** (0.437 µM), and **3a** (0.455 µM), indicating a wide therapeutic potential. In contrast, tris(4-fluorophenyl)phosphine containing **3d** derivative displayed more moderate efficacy across most panels (mean *GI*_50_: 2.046 µM). However, except for compound **3d**, all hybrids consistently demonstrated submicromolar mean activity across multiple tumor types. The strongest effect was represented by phosphonium salt **3f** on the K-562 leukemia cell line (*GI*_50_ = 34 nM), but values below 100 nM were also achieved on the leukemia HL-60(TB) (78 nM), CNS cancer U251 (67 nM), and breast cancer MDA-MB-468 (64 nM) cell lines.

A detailed comparison of the phosphine substituents (**1a**–**j**) reveals preliminary structure–activity correlations among the chrysin–phosphine hybrids (**3a**–**j**). The nature of the phosphine group plays a critical role in modulating antiproliferative potency. Electron-donating aryl phosphines, such as those found in **1f** (heavily methylated and methoxylated aryl), **1b** (tri-*p*-tolyl), and **1g** (tri-*o*-tolyl), resulted in the most potent hybrids (**3f**, **3b**, and **3g**), with mean *GI*_50_ values of 0.312 µM, 0.322 µM, and 0.437 µM, respectively. These groups are likely to enhance membrane permeability and facilitate stronger intracellular interactions due to increased lipophilicity and π-electron density. On the contrary, moderate potency was observed for fluorinated aryl phosphonium derivative **3d** and the pyridine-containing hybrid **3h**, likely due to electron-withdrawing effects. Aliphatic tricyclohexylphosphine **1e** consistently led to diminished activity across assays. This can be attributed to their lack of aromaticity and reduced ability to engage in π-stacking or electronic interactions with biological targets. In summary, the most favorable activity profiles were associated with electron-rich arylphosphines. These findings suggest that consideration of electronic properties is essential for optimizing the anticancer potential of chrysin–phosphine hybrids. The results suggest that both **3f** and **3b** are the most promising candidates for further development, combining potent cytotoxicity with broad-spectrum efficacy.

#### 2.2.2. Antiproliferative Properties of Compounds **3i**, **3j**, and **5**

As the NCI has not been accepting compound submissions from investigators outside the USA for NCI-60 testing since 2024, chrysin derivatives **3i, 3j**, and **5** were instead evaluated in additional viability assays against a simplified panel of human cancer cell lines representing breast (MCF-7 and MDA-MB-231), cervical (HeLa and SiHa), and ovarian cancers. A nonmalignant fibroblast cell line (NIH/3T3) was also used to obtain preliminary results describing the cancer selectivity of the test substances. Regarding the cell growth-inhibiting effect of the tested chrysin derivatives, the two phosphonium analogs (**3i**, **3j**) exhibited outstanding effects, with inhibition rates exceeding 80% even at 10 µM (Table 2). As expected, substitution of the phosphonium element with triphenylpropanoic acid resulted in an ineffective compound (**5**). The two effective agents were further investigated using a broad concentration range (0.1–30 µM), and their *IC*_50_ values were calculated (Table 3). According to the obtained results, both compounds exhibit a more pronounced antiproliferative effect than the reference agent cisplatin, and **3i** is more potent, with *IC*_50_ values in the 0.26–1.15 µM range, while those of **3j** are between 0.88 and 2.93 µM.

Besides the cell growth-inhibiting potency, cancer selectivity is a crucial factor determining the perspective of a drug candidate. This property is expressed as a selectivity ratio, i.e., the ratio of the compound’s *IC*_50_ obtained on the non-cancerous fibroblasts and those from the cancer cell lines. A higher ratio indicates more promising cancer selectivity (Table 3). **3i** proved to be more selective than **3j** and cisplatin, with the ratios of the agents ranging from 1.44 to 6.38, 0.67 to 2.23, and 0.14 to 1.93, respectively. Based on these efficacy and selectivity data, further cell-based studies were performed with **3i** against MCF-7 breast cancer cells.

#### 2.2.3. Cell Cycle Analysis

**Figure 3:**
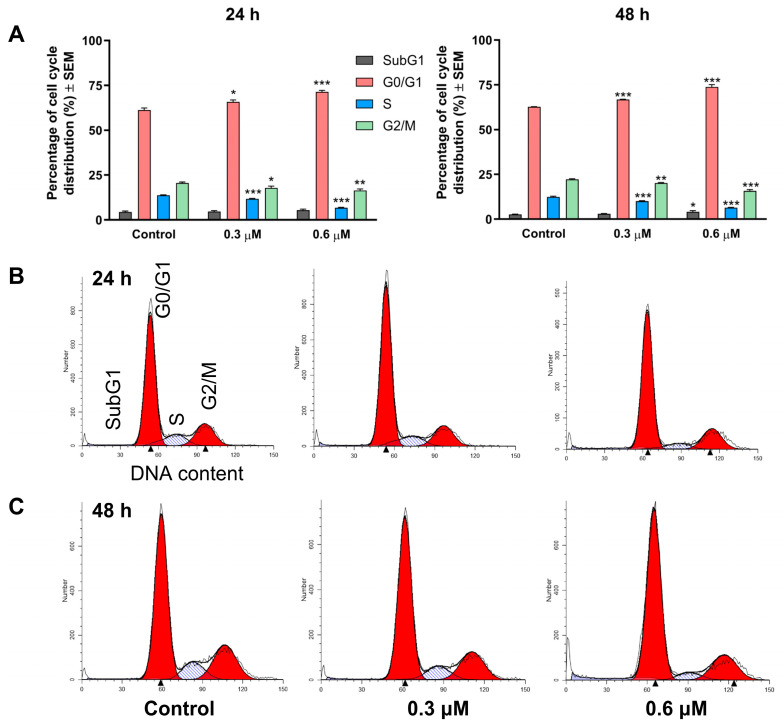
The effect of derivative **3i** on the cell cycle distribution of MCF-7 breast cancer cells after 24 or 48 h of incubation (**A**). *, ** and *** indicate significance at *p* < 0.05, *p* < 0.01 and *p* < 0.001, respectively, compared to the control. Representative flow cytometry histograms recorded after 24 (**B**) and 48 (**C**) hours of treatment.

Treatment of MCF-7 cells with **3i** resulted in a concentration- and time-dependent alteration of the cell cycle. At 24 h, the compound (0.3 and 0.6 µM) significantly increased the proportion of the G0/G1 cell population at the expense of cells in the synthetic (S) and G2/M phases (Figure 3). In the case of prolonged exposure (48 h), these alterations became even more pronounced, and a modest but statistically significant accumulation of the hypodiploid (SubG1) cell population was also observed. The observed cell cycle disturbance is consistent with a blockade at the G1–S transition, which triggers apoptotic cell death.

#### 2.2.4. Antimigratory Property

**Figure 4:**
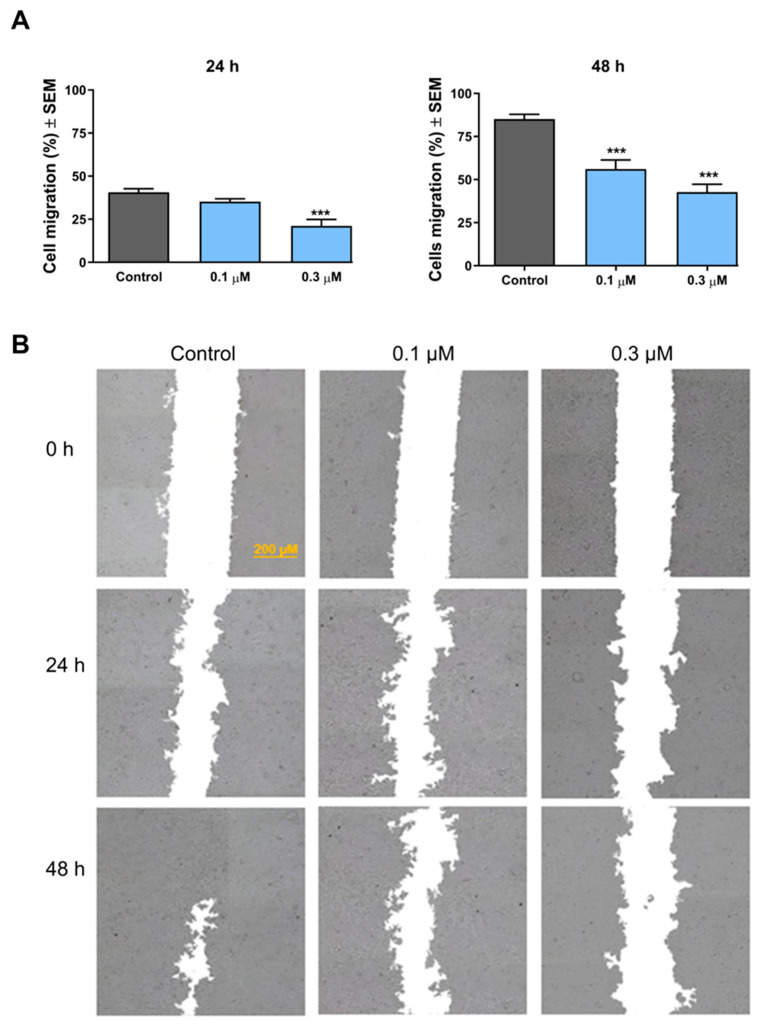
The effect of **3i** on the migration capacity of MCF-7 cells. The bar charts show the percentage of cell-free areas after 24 and 48 h of treatment (**A**). *** indicates significance at *p* < 0.001 compared to the control condition. Representative images of reduced wound healing at 0, 24, and 48 h post-treatment (**B**).

The effect of the selected compound on the migration of MCF-7 cells was investigated by means of the wound-healing assay. According to the results, **3i** exhibited a concentration- and incubation time-dependent inhibition of the migration of the treated cancer cells (Figure 4). After a short exposure (24 h), the compound exerted significant inhibition in the migration capacity at a concentration of 0.3 μM only. After 48 h of treatment, however, a more pronounced inhibition was recorded, which was significant even at subantiproliferative concentration (0.1 μM).

#### 2.2.5. CellTiter-Glo Luminescent Cell Viability Assay on Non-Tumor CHO Cells

**Figure 5:**
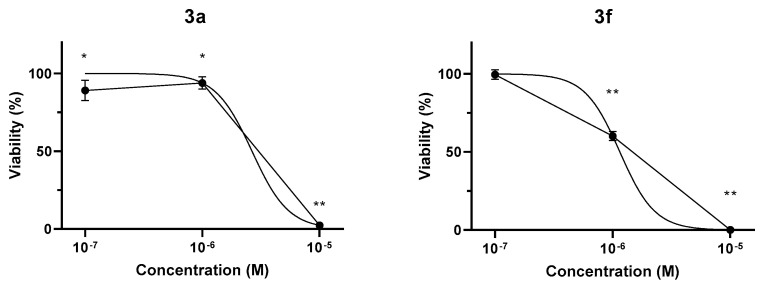
The effect of **3a** and **3f** on the viability of CHO cells. Normalized viability values following 48 h treatment are represented as means ± SD of 3 independent experiments in technical triplicates (*n* = 9); one-way ANOVA + Dunnett’s multiple comparison test, * *p* < 0.01, ** *p* < 0.0001 vs. control. Non-linear regression curves were fitted to the measured values to determine *IC*_50_.

Given their potent antiproliferative effects on tumor cells, two conjugates were selected for testing on non-tumor CHO cells to investigate their selectivity. Following 48 h treatment with **3a** and **3f** (10^−7^–10^−5^ M), CellTiter-Glo Luminescent Cell Viability Assay showed a concentration-dependent decrease in the luminescent signal, reflecting attenuation of cell viability. **3a** minimally affected non-tumor cell viability up to 10^−6^ M concentration, while the 10^−6^ M concentration of **3f** decreased cell viability to 60.2 ± 2.9%. Treatment with either conjugate resulted in a total reduction of CHO cell viability at 10^−7^ M concentration (Figure 5). Estimated *IC*_50_ values were 2.65 µM and 1.15 µM for **3a** and **3f**, respectively. Since significant inhibitory effects of the conjugates were observed only at substantially higher concentrations in non-tumor CHO cells compared to most of the investigated tumor cell lines, these compounds appear to exhibit promising tumor cell selectivity.

## 3. Materials and Methods

### 3.1. General

Chrysin, 1,4-dibromobutane, triphenylphosphine (TPP), tri(*p*-tolyl)phosphine, tris(4-methoxyphenyl)phosphine, tris(4-fluorophenyl)phosphine, tricyclohexyl-phosphine, tris(4-methoxy-3,5-dimethylphenyl)phosphine, tri(*o*-tolyl)phosphine, diphenyl-2-pyridylphosphine, diphenyl(2-methoxyphenyl)phosphine, *tert*-butyldiphenylphosphine, 3,3,3-triphenylpropionic acid, thionyl chloride (SOCl_2_), and triethylamine (NEt_3_, TEA) were obtained from Merck (Budapest, Hungary). Potassium carbonate (K_2_CO_3_), acetone, toluene, acetonitrile (ACN), dichloromethane (DCM), dimethylformamide (DMF), and methanol (MeOH) were purchased from Molar Chemicals Ltd. (Budapest, Hungary). Melting points were measured on a VEB Analytik Dresden PHMK-77/1328 apparatus (Dresden, Germany) and are uncorrected.

NMR measurements were performed on a Bruker Avance III HDX 400 MHz NMR spectrometer equipped with a ^31^P–^15^N{1H–^19^F} 5 mm CryoProbe Prodigy BBO probe, a Bruker Avance III HDX 500 MHz NMR spectrometer equipped with a ^1^H{^13^C/^15^N} 5 mm TCI CryoProbe, a Varian VNMRS 600 MHz NMR System NMR spectrometer, and a Bruker Avance III HDX 800 MHz NMR spectrometer equipped with a ^1^H–^19^F{^13^C/^15^N} 5 mm TCI CryoProbe (Bruker Corporation, Billerica, MA, USA). ^1^H and ^13^C chemical shifts are given on the delta scale as parts per million (ppm) relative to tetramethyl silane. One-dimensional ^1^H and ^13^C spectra and two-dimensional ^1^H–^1^H COSY, ^1^H–^1^H NOESY, ^1^H–^13^C HSQC, and ^1^H–^13^C HMBC spectra were acquired using pulse sequences included in the standard spectrometer software packages (Bruker TopSpin 3.5, Bruker Corporation; VNMRJ 3.2, Agilent Technologies, Santa Clara, CA, USA). NMR spectra were processed with Bruker TopSpin 3.5 pl 6 (Bruker Corporation, Billerica, MA, USA) and ACD/Spectrus Processor version 2017.1.3 (Advanced Chemistry Development, Inc., Toronto, ON, Canada).

ESI-HRMS and MS-MS analyses were performed on a Thermo Velos Pro Orbitrap Elite (Thermo Fisher Scientific, Bremen, Germany) system. The ionization method was ESI, operated in positive ion mode. The protonated molecular ion peaks were fragmented by CID (collision-induced dissociation) at a normalized collision energy of 40–45%. For the CID experiment, helium was used as the collision gas. Data acquisition and analysis were accomplished with Xcalibur software version 4.0 (Thermo Fisher Scientific).

The reactions were followed by analytical thin layer chromatography (TLC) on DC-Alufolien Kieselgel 60 F_254_ (Merck, Budapest, Hungary) plates. Preparative TLC analyses were performed on silica gel 60 PF_254+366_ (Merck) glass plates. Column chromatography was carried out using Silica gel 60 (0.040–0.063 mm) (Merck).

### 3.2. Chemistry

A detailed description of the syntheses can be found in the Supplementary Materials. The NMR assignments and HRMS data of the new derivatives (**3a–j** and **5**) are also given in the Supplementary Materials, as well as the skeleton numberings of compounds used for NMR assignment (Figures S1–S11).

### 3.3. Biological Evaluation

#### 3.3.1. NCI60 Screening

A detailed description of the NCI screening procedures [36,37,38], including the one-dose and five-dose tests, can be found in the Supplementary Materials, on the website of NCI [39], and in our previous work [26].

#### 3.3.2. Determination of Antiproliferative Effects on Adherent Cancer Cell Lines

The cell growth-inhibiting properties of compounds **3i**, **3j**, and **5** were determined by means of the MTT assay against a panel of human malignant cell lines of gynecological origin [8,27,40]. The panel included breast (MCF-7 and MDA-MB-231; Cellosaurus accessions: CVCL_0031 and CVCL_0062, respectively), cervical (HeLa and SiHa; CVCL_0030 and CVCL_0032, respectively), and ovarian (A2780; CVCL_0134) cell lines. Nonmalignant murine NIH/3T3 (CVCL_0594) fibroblasts were also used to obtain results characterizing the cancer selectivity of the tested substances. The whole assay was performed as described earlier [8]. The presented analogs were initially tested at two concentrations (10 and 30 μM), and the assays were repeated with a broader concentration range (0.1–30 μM) to calculate the *IC*_50_ values for more potent compounds using GraphPad Prism 10.6.1 software (GraphPad Software, San Diego, CA, USA). All applied cell lines were obtained from the European Collection of Cell Cultures (Salisbury, UK), except for SiHa (American Tissue Culture Collection, Manassas, VA, USA). Cisplatin, a clinically available anticancer drug, was included as a reference compound. All experiments were conducted twice, with at least five replicates per condition.

#### 3.3.3. Cell Cycle Analysis

Cell cycle analysis by flow cytometry was applied to characterize the effects of **3i** on the phase distribution of the treated cells. MCF-7 cells were seeded onto 12-well plates (200,000 cells/well) and incubated overnight. They were then treated with 0.3 or 0.6 μM for 24 or 48 h. After the treatment, the propidium iodide-labeled cells were analyzed using a CytoFLEX instrument (Beckman Coulter, Brea, CA, USA), and at least 20,000 cells per sample were evaluated, as described in a recent publication [8]. The obtained data were processed using ModFit LT 3.3.11 software (Verity Software House, Topsham, ME, USA) as referenced by Vermes et al. [41].

#### 3.3.4. Determination of Antimigratory Effect

The effect of substance **3i** on the migration of the treated cancer cells was determined using a wound-healing assay, as presented earlier [8,42]. Briefly, MCF-7 cells were trypsinized and plated into wound assay chambers (ibidi GmbH, Martinsried, Germany) at a density of 40,000 cells/insert, and the plate was incubated under standard conditions to allow proper attachment. Then, the inserts were carefully removed, and the cells were subsequently treated with **3i** in fresh medium containing 2% FBS. The cell migration was recorded using a phase-contrast inverted microscope (Nikon Instruments Europe, Amstelveen, The Netherlands) fitted with a CCD camera (QImaging MicroPublisher Color RTV5.0 Teledyne Photometrics, Tucson, AZ, USA). The cell-free areas in treated samples and controls were determined using ImageJ software 1.54d (National Institutes of Health, Bethesda, MD, USA). The assays were performed in triplicates and all the statistical analyses were carried out by means of GraphPad Prism software.

#### 3.3.5. CellTiter-Glo Luminescent Cell Viability Assay on Non-Tumor CHO Cells

Cell viability of non-tumor CHO cells was assessed using the CellTiter-Glo Luminescent Cell Viability Assay (Promega, Madison, WI, USA), following treatment with **3a** and **3f [43,44,45,46,47,48]**. Stock solutions (10 mM in DMSO) were stored frozen until use. CHO cells were cultured in complete DMEM (low glucose, 1 g/L) supplemented with 10% fetal bovine serum, 1% GlutaMAX-I, 1% non-essential amino acids, and 0.1% penicillin–streptomycin. Cells were seeded into opaque 96-well plates (5000 cells/100 µL/well), with peripheral wells filled with sterile PBS to minimize edge effects. After overnight incubation (37 °C, 5% CO_2_), cells were treated with increasing concentrations of the compounds (10^−5^, 10^−6^, and 10^−7^ M) diluted in complete DMEM. Untreated cells served as controls, and complete DMEM was used to determine background luminescence. DMSO controls corresponding to the solvent concentrations of each treatment condition were also included; no significant effect on cell viability was observed. After 48 h, CellTiter-Glo reagent was added, and luminescence was measured using an EnSpire AlphaLisa microplate reader (Perkin Elmer). Relative light unit (RLU) values were normalized to untreated controls (100% viability). Non-linear regression analysis was performed in GraphPad Prism 8.0.1 to estimate IC_50_ values by fitting sigmoidal dose–response curves (log[inhibitor] vs. normalized response, variable slope).

## 4. Conclusions

In this study, we reported the synthesis and biological evaluation of novel phosphonium derivatives of chrysin. These hybrids contain chrysin conjugated to trisubstituted (mainly triaryl) phosphines through a 4-carbon linker at position 7. The compounds presumably exert their anticancer effects via mitochondrial targeting, facilitated by triarylphosphines bearing electron-donating substituents that stabilize the positively charged phosphorus center. The prepared phosphonium salts (**3a**–**j**) induced significant cytotoxicity in several cancer cell lines. Among them, hybrid **3i** caused cell-cycle blockade and inhibited breast cancer cell migration even at subantiproliferative concentrations. In contrast, a neutral trityl analog (**5**) showed no cytotoxicity. The two most potent derivatives were the tri(*p*-tolyl) (**3b**) and tris(4-methoxy-3,5-dimethylphenyl) (**3f**) hybrids, with mean *GI*_50_ values of 0.322 and 0.312 µM, respectively. These findings are consistent with our previous work on vindoline–phosphine conjugates, where the same phosphine types (**1b** and **1f**) were the most effective. Notably, **3a** and **3f** displayed cytotoxicity toward non-tumor CHO cells only at much higher concentrations (*IC*_50_ = 2.65 and 1.15 µM, respectively), suggesting tumor-selective activity—a promising feature for further anticancer development.

## Figures and Tables

**Table 1:** *GI*_50_ values (µM) of compounds **3a***–***h** against 60 human cancer cell lines in vitro. Values where *GI*_50_ < 0.5 µM are highlighted in bold. n.d. means not determined.

		3a		3b		3c		3d		3e		3f		3g		3h	
Panel	Cell Line	GI_50_	Mean	GI_50_	Mean	GI_50_	Mean	GI_50_	Mean	GI_50_	Mean	GI_50_	Mean	GI_50_	Mean	GI_50_	Mean
Leukemia	CCRF-CEM	**0.335**	**0.26**	**0.324**	**0.22**	**0.367**	**0.34**	2.310	1.71	**0.477**	**0.42**	**0.103**	**0.12**	**0.242**	**0.21**	**0.334**	**0.26**
	HL-60(TB)	**0.167**		**0.140**		**0.288**		1.340		**0.184**		**0.078**		**0.180**		**0.130**	
	K-562	**0.216**		**0.137**		**0.376**		1.400		**0.402**		**0.034**		**0.279**		**0.189**	
	MOLT-4	**0.338**		**0.322**		**0.453**		1.900		1.010		**0.238**		**0.235**		**0.424**	
	RPMI-8226	**0.205**		**0.144**		**0.235**		1.310		**0.182**		**0.110**		**0.135**		**0.248**	
	SR	**0.289**		**0.261**		**0.300**		1.990		**0.260**		**0.162**		**0.206**		**0.214**	
Non-small-Cell Lung Cancer	A549/ATCC	0.908	**0.46**	**0.393**	**0.31**	0.600	0.59	3.210	1.97	1.330	1.07	**0.314**	**0.29**	**0.417**	**0.45**	1.130	1.07
	EKVX	**0.317**		**0.275**		**0.462**		2.230		1.060		**0.309**		n.d.		1.120	
	HOP-62	**0.480**		**0.307**		0.732		1.560		1.560		**0.311**		**0.332**		1.520	
	HOP-92	**0.234**		**0.163**		**0.252**		1.320		**0.211**		**0.165**		**0.255**		**0.346**	
	NCI-H226	**0.354**		**0.277**		0.606		1.820		1.100		**0.249**		**0.399**		1.560	
	NCI-H23	**0.302**		**0.241**		**0.423**		1.650		1.340		**0.283**		**0.243**		0.776	
	NCI-H322M	0.976		0.532		1.420		1.990		1.580		**0.432**		1.380		2.040	
	NCI-H460	**0.321**		**0.357**		**0.371**		2.130		0.957		**0.304**		**0.243**		0.801	
	NCI-H522	**0.228**		**0.274**		**0.405**		1.820		**0.457**		**0.221**		**0.341**		**0.320**	
Colon Cancer	COLO 205	1.020	0.62	**0.419**	**0.39**	**0.455**	0.64	1.620	1.69	0.812	0.94	0.791	**0.41**	**0.351**	0.58	1.640	1.07
	HCC-2998	**0.345**		**0.380**		**0.300**		1.670		0.504		**0.297**		**0.239**		**0.445**	
	HCT-116	**0.297**		**0.248**		**0.337**		1.670		0.882		**0.256**		**0.265**		0.650	
	HCT-15	0.628		**0.311**		1.550		1.850		1.550		**0.345**		1.410		1.580	
	HT29	1.280		0.855		1.250		1.840		1.770		0.646		1.220		1.750	
	KM12	**0.401**		**0.160**		**0.207**		1.700		**0.382**		**0.195**		**0.280**		0.523	
	SW-620	**0.343**		**0.354**		**0.351**		1.460		0.649		**0.323**		**0.274**		0.901	
CNS Cancer	SF-268	**0.380**	**0.30**	**0.285**	**0.30**	**0.351**	**0.46**	1.570	1.67	1.040	0.75	0.525	**0.28**	**0.279**	**0.33**	0.760	0.77
	SF-295	**0.297**		**0.215**		1.060		1.520		1.400		**0.240**		0.648		1.360	
	SF-539	**0.380**		**0.498**		**0.410**		1.930		0.751		**0.367**		**0.187**		1.060	
	SNB-19	**0.279**		**0.295**		**0.333**		1.670		0.505		**0.276**		**0.353**		0.524	
	SNB-75	**0.165**		**0.219**		**0.344**		1.670		0.529		**0.213**		**0.274**		0.572	
	U251	**0.315**		**0.279**		**0.276**		1.670		0.287		**0.067**		**0.225**		**0.335**	
Melanoma	LOX IMVI	**0.326**	**0.30**	**0.287**	**0.32**	**0.355**	**0.35**	1.690	1.74	0.938	0.55	**0.284**	**0.31**	**0.170**	**0.27**	0.694	0.52
	MALME-3M	**0.270**		**0.381**		**0.287**		2.040		**0.397**		**0.310**		**0.353**		0.594	
	M14	**0.378**		**0.331**		**0.478**		1.750		0.515		**0.329**		**0.335**		0.564	
	MDA-MB-435	**0.299**		**0.238**		**0.319**		1.550		**0.341**		**0.296**		**0.320**		**0.376**	
	SK-MEL-2	**0.238**		**0.246**		**0.301**		1.730		1.320		**0.292**		**0.194**		0.758	
	SK-MEL-28	**0.474**		0.514		**0.453**		1.690		**0.423**		**0.491**		**0.323**		0.570	
	SK-MEL-5	**0.166**		**0.153**		**0.331**		1.510		**0.369**		**0.168**		**0.198**		**0.278**	
	UACC-257	**0.282**		**0.367**		**0.277**		1.660		**0.267**		**0.298**		**0.261**		**0.298**	
	UACC-62	**0.292**		**0.344**		**0.317**		2.050		**0.368**		**0.312**		**0.319**		0.549	
Ovarian Cancer	IGROV1	**0.405**	**0.89**	**0.416**	**0.48**	**0.413**	0.76	1.890	3.90	0.774	1.95	**0.346**	0.51	**0.428**	0.82	0.653	1.89
	OVCAR-3	**0.368**		**0.196**		**0.288**		1.420		**0.381**		**0.334**		**0.216**		**0.418**	
	OVCAR-4	**0.370**		**0.247**		**0.382**		1.640		0.686		**0.351**		**0.429**		1.140	
	OVCAR-5	1.150		0.684		0.852		3.000		1.300		**0.335**		1.720		1.440	
	OVCAR-8	**0.350**		**0.337**		**0.408**		1.840		0.595		**0.350**		0.560		**0.494**	
	NCI/ADR-RES	3.290		1.180		2.600		15.900		8.440		1.580		2.140		7.550	
	SK-OV-3	**0.329**		**0.301**		**0.382**		1.620		1.470		**0.263**		**0.277**		1.520	
Renal Cancer	786-0	0.553	0.53	**0.373**	**0.31**	1.210	1.03	1.830	2.10	1.570	1.30	**0.338**	**0.32**	**0.291**	0.58	1.570	1.35
	A498	**0.422**		**0.400**		1.570		2.070		1.690		**0.367**		**0.462**		1.730	
	ACHN	0.633		**0.265**		1.280		3.230		1.520		**0.371**		0.569		1.640	
	CAKI-1	**0.385**		**0.228**		1.030		1.780		1.570		**0.275**		**0.309**		1.540	
	RXF 393	**0.225**		**0.333**		**0.257**		1.820		**0.461**		**0.191**		n.d.		0.608	
	SN12C	**0.330**		**0.389**		**0.317**		2.490		0.509		**0.313**		**0.230**		0.510	
	TK-10	**0.464**		**0.313**		1.090		1.740		1.430		**0.427**		**0.414**		1.690	
	UO-31	1.240		**0.177**		1.480		1.850		1.610		**0.277**		1.770		1.540	
Prostate Cancer	PC-3	**0.305**	**0.41**	**0.182**	**0.20**	**0.320**	**0.37**	1.760	1.74	0.501	0.87	**0.276**	**0.31**	**0.308**	**0.34**	0.572	0.90
	DU-145	0.507		**0.223**		**0.410**		1.720		1.240		**0.339**		**0.376**		1.220	
Breast Cancer	MCF7	**0.291**	**0.24**	**0.268**	**0.25**	**0.407**	**0.33**	1.620	1.61	0.956	0.59	**0.260**	**0.22**	**0.267**	**0.25**	**0.396**	0.53
	MDA-MB231ATCC	**0.240**		**0.320**		**0.315**		1.910		**0.366**		**0.258**		**0.337**		0.906	
	HS 578T	**0.306**		**0.366**		**0.332**		1.980		0.838		**0.361**		**0.232**		1.130	
	BT-549	**0.257**		**0.344**		**0.349**		1.610		0.709		**0.280**		**0.291**		**0.293**	
	T-47D	**0.228**		**0.105**		**0.302**		1.450		**0.397**		**0.121**		**0.208**		**0.259**	
	MDA-MB-468	**0.128**		**0.123**		**0.248**		1.100		**0.273**		**0.064**		**0.148**		**0.176**	
Mean		**0.455**		**0.322**		0.565		2.046		0.957		**0.312**		**0.437**		0.949	

**Table 2:** The antiproliferative properties of the selected phosphonium derivatives.

Cell Line	Conc. (μM)	Growth Inhibition (%) ± SEM		
		3i	3j	5
HeLa	10 30	96.46 ± 2.76 98.87 ± 0.86	96.64 ± 0.26 97.55 ± 0.65	>10 * >10
SiHa	10 30	83.15 ± 2.13 103.50 ± 0.41	97.91 ± 0.06 97.81 ± 0.46	>10 >10
MCF-7	10 30	95.40 ± 1.54 95.82 ± 1.45	98.65 ± 0.29 98.68 ± 0.32	>10 >10
MDA-MB-231	10 30	88.58 ± 3.72 87.43 ± 2.89	97.98 ± 1.03 98.10 ± 0.13	>10 >10
A2780	10 30	96.85 ± 0.37 98.26 ± 0.87	98.77 ± 0.19 98.96 ± 0.38	>10 >10
NIH/3T3 (fibroblast)	10 30	97.73 ± 0.53 98.52 ± 0.95	98.38 ± 0.53 99.00 ± 0.10	>10 >10

* Growth inhibition values less than 10% are considered negligible and not presented numerically.

**Table 3:** The calculated *IC*_50_ values and tumor-selectivity ratios of compounds **3i**, **3j**, and cisplatin.

Cell Line	IC_50_ Value (μM) ± SEM		
	Selectivity Ratio		
	3i	3j	Cisplatin *
HeLa	1.08 ± 0.02 1.54	2.93 ± 0.02 0.67	13.60 ± 0.44 0.20
SiHa	1.15 ± 0.03 1.44	1.47 ± 0.01 1.33	15.50 ± 0.93 0.17
MCF-7	0.26 ± 0.02 6.38	0.88 ± 0.01 2.23	2.60 ± 0.17 1.04
MDA-MB-231	0.54 ± 0.02 3.07	1.24 ± 0.02 1.58	19.13 ± 0.12 0.14
A2780	0.66 ± 0.03 2.52	1.37 ± 0.04 1.43	1.40 ± 0.11 1.93
NIH/3T3 (fibroblast)	1.66 ± 0.15	1.96 ± 0.01	2.70 ± 0.30

* Cisplatin *IC*_50_ values have been published earlier [8].

## Data Availability

The original contributions presented in this study are included in the article/Supplementary Material. Further inquiries can be directed to the corresponding authors.

## Supplementary Materials

The following supporting information can be downloaded at: https://www.mdpi.com/article/10.3390/ijms262211063/s1.
