# Supplementary material for: Synthesis and In Vitro Anticancer Evaluation of Novel Phosphonium Derivatives of Chrysin
Source: Int J Mol Sci. 2025 Nov 15;26(22):11063. doi: 10.3390/ijms262211063 (PMC12652692; doi:10.3390/ijms262211063)
Supplement: Supplementary file 1 [file ijms-26-11063-s001.zip › ijms-3980332-supplementary.pdf]

International Journal of
Molecular Sciences

MDPI

Article

Synthesis and In Vitro Anticancer Evaluation of Novel Phosphonium Derivatives of Chrysin

Mónika Halmai [1], Dominika Mária Herr [1], Szabolcs Mayer [1], Péter Keglevich [1*], Ejjal A. Abdallah [2], Noémi Bózsity-Faragó [2], István Zupkó [2], Andrea Nehr-Majoros [3,4,5], Éva Szőke [3,4,5], Zsuzsanna Helyes [3,4,5,6] and László Hazai [1*]

[1] Department of Organic Chemistry and Technology, Faculty of Chemical Technology and Biotechnology, Budapest University of Technology and Economics, Műegyetem rkp. 3, H-1111 Budapest, Hungary, monika.halmai@edu.bme.hu (M.H.)
[2] Institute of Pharmacodynamics and Biopharmacy, University of Szeged, Eötvös u. 6, H-6720 Szeged, Hungary
[3] Department of Pharmacology and Pharmacotherapy, Medical School & Centre for Neuroscience, University of Pécs, Szigeti út 12, H-7624 Pécs, Hungary
[4] National Laboratory for Drug Research and Development, Magyar Tudósok krt. 2, H-1117 Budapest, Hungary
[5] HUN-REN PTE Chronic Pain Research Group, Szigeti út 12, H-7624 Pécs, Hungary
[6] PharmInVivo Ltd., Szondi Gy. u. 10, H-7629 Pécs, Hungary
* Correspondence: keglevich.peter@vbk.bme.hu, hazai.laszlo@vbk.bme.hu

Supplementary Materials

S.1. Chemistry

S.1.1. Preparation of 7-(4-bromobutoxy)chrysin (4)

^{1}H NMR, m.p., and R_f data were in good agreement with the literature [24,35].

S.1.2. Synthesis of chrysin - phosphine conjugates (3a–3j)

S.1.2.1. Preparation of (4-((5-hydroxy-4-oxo-2-phenyl-4*H*-chromen-7-yl)oxy)butyl)triphenylphosphonium bromide (3a)

7-(4-Bromobutoxy)chrysin (**4**) (150 mg, 0.385 mmol) and triphenylphosphine (**1a**) (303 mg, 1.156 mmol, 3 eq.) were added to a pressure-resistant vessel and dissolved in anhydrous toluene (7 mL). The reaction mixture was stirred at 110 °C under an Ar atmosphere. After a total reaction time of 38 hours, the reaction mixture was evaporated under reduced pressure. The crude product was purified by preparative TLC (DCM:MeOH = 10:1) to afford the hybrid molecule **3a** as a solid (173 mg, 69%). M.p.: 96–98 °C. TLC (DCM:MeOH = 5:1); R_f = 0.60. ^{1}H NMR (499.9 MHz; $CDCl_3$) δ (ppm) 1.79–1.96 (2H; m; H_2-2'); 2.25–2.33 (2H; m; H_2-3'); 3.93–4.02 (2H; dm; *J*= 13.1 Hz; H_2-1'); 4.22 (2H; t; *J* = 5.6 Hz; H_2-4'); 6.19 (1H; d; *J* = 2.0 Hz; H-6); 6.57 (1H; d; *J* = 2.0 Hz; H-8); 6.66 (1H; s; H-3); 7.50–7.57 (3H; m; C(2)-Ph: H_{meta}, H_{para}); 7.66–7.72 (6H; m; P-Ph: H_{meta}); 7.77–7.81 (3H; m; P-Ph: H_{para}); 7.83–7.89 (6H; dm; *J* = 12.6 Hz; P-Ph: H_{orto}); 7.89–7.92 (2H; m; C(2)-Ph: H_{orto}); 12.67 (1H; s; C(5)-O<u>H</u>). ^{13}C NMR (125.7 MHz; $CDCl_3$) δ (ppm) 19.1 (d; *J* = 3.8 Hz; C-2'); 21.9 (d; *J* = 50.7 Hz; C-1'); 28.9 (d; *J* = 16.9 Hz; C-3'); 67.2 (C-4'); 92.6 (C-8); 99.3 (C-6); 105.77 (C-3); 105.84 (C-10); 118.3 (d; *J* = 85.8 Hz; P-Ph: C_{ipszo}); 126.4 (C(2)-Ph: C_{orto}); 129.1 (C(2)-Ph: C_{meta}); 130.5 (d; *J* = 12.6 Hz; P-Ph: C_{meta}); 131.1 (C(2)-Ph: C_{ipszo}); 131.9 (C(2)-Ph: C_{para}); 133.7 (d; *J* = 9.9 Hz; P-Ph: C_{orto}); 135.0 (d; *J* = 2.9 Hz; P-Ph: C_{para}); 158.0 (C-9); 161.7 (C-5); 164.1 (C-2); 164.8 (C-7); 182.5 (C-4). HRMS: M^+=571.20341 (delta=0.2 ppm; $C_{37}H_{32}O_4P$), HR-ESI-MS-MS (CID=60%; rel, int, %): 317(100); 309(6); 289(64); 275(16); 267(21); 262(27); 255(6); 199(5).

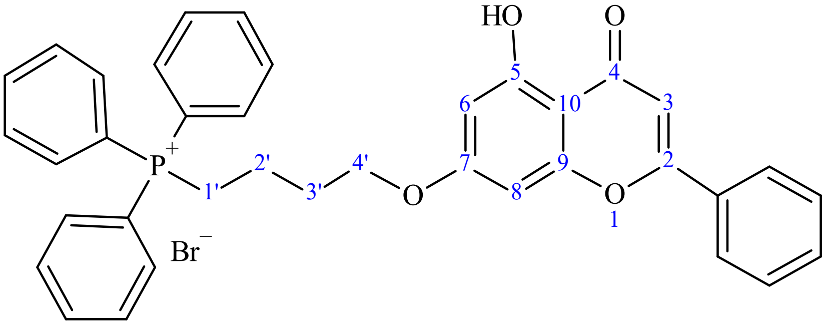

Figure S1. The skeleton numbering of compound **3a** used for NMR assignment.

S.1.2.2. Preparation of (4-((5-hydroxy-4-oxo-2-phenyl-4*H*-chromen-7-yl)oxy)butyl)tri-p-tolylphosphonium bromide (3b)

7-(4-Bromobutoxy)chrysin (**4**) (100 mg, 0.257 mmol) and tri-*p*-tolylphosphine (**1b**) (118 mg, 0.389 mmol, 1.5 eq.) were added to a round-bottom flask and dissolved in anhydrous toluene (5 mL). The reaction mixture was refluxed under an Ar atmosphere. After 12 hours, an additional 80 mg (0.262 mmol, 1.0 eq.) of tri-*p*-tolylphosphine (**1b**) was added. After a total reaction time of 38 hours, the reaction mixture was evaporated under reduced pressure, washed multiple times with tert-butyl methyl ether, and filtered through a glass filter. The crude product was purified by preparative TLC (DCM:MeOH = 10:1) to afford the hybrid molecule **3b** as a solid (128 mg, 72%). M.p.: 88–90 ◦C. TLC (DCM:MeOH = 10:1); R_f = 0.37. ^{1}H NMR (499.9 MHz; DMSO-d_6) δ (ppm) 1.63-1.75 (2H; m; H_2-3′); 1.94 (2H; qui; *J* = 6.6 Hz; H_2-2′); 2.44 (9H; s; 3×P-Ph-C<u>H</u>$_3$); 3.50-3.60 (2H; m; H_2-4′); 4.18 (2H; t; *J* = 6.2 Hz; H_2-1′); 6.34 (1H; d; *J* = 2.2 Hz; H-6); 6.77 (1H; d; *J* = 2.2 Hz; H-8); 7.06 (1H; s; H-3); 7.53-7.69 (15H; m; 2×C(2)-Ph: H_{meta}, C(2)-Ph: H_{para}, 6×P-Ph: H_{orto}, 6×P-Ph: H_{meta}); 8.08-8.13 (2H; m; 2×C(2)-Ph: H_{orto}); 12.81 (1H; s; C(5)-O<u>H</u>). ^{13}C NMR (125.7 MHz; DMSO-d_6) δ (ppm) 18.1 (d; *J* = 3.4 Hz; C-3′); 19.8 (d; *J* = 52.2 Hz; C-4′); 21.1 (d; *J* = 1.1 Hz; 3×P-Ph-<u>C</u>H$_3$); 28.7 (d; *J* = 16.9 Hz; C-2′); 66.9 (C-1′); 93.3 (C-8); 98.4 (C-6); 104.8 (C-10); 105.3 (C-3); 115.3 (d; *J* = 88.3 Hz; 3×P-Ph: C_{ipszo}); 126.3 (2×C(2)-Ph: C_{orto}); 129.1 (2×C(2)-Ph: C_{meta}); 130.5 (C(2)-Ph: C_{ipszo}); 130.7 (d; *J* = 12.9 Hz; 6×P-Ph: C_{meta}); 132.1 (C(2)-Ph: C_{para}); 133.3 (d; *J* = 10.5 Hz; 6×P-Ph: C_{orto}); 145.5 (d; *J* = 2.9 Hz; 3×P-Ph: C_{para}); 157.2 (C-9); 161.1 (C-5); 163.4 (C-2); 164.6 (C-7); 182.0 (C-4). HRMS: M^+=613.24894 (delta=-2.1 ppm; $C_{40}H_{38}O_4P$), HR-ESI-MS-MS (CID=40%; rel, int, %): 359(100); 331(58); 317(14); 304(28); 267(6); 227(4).

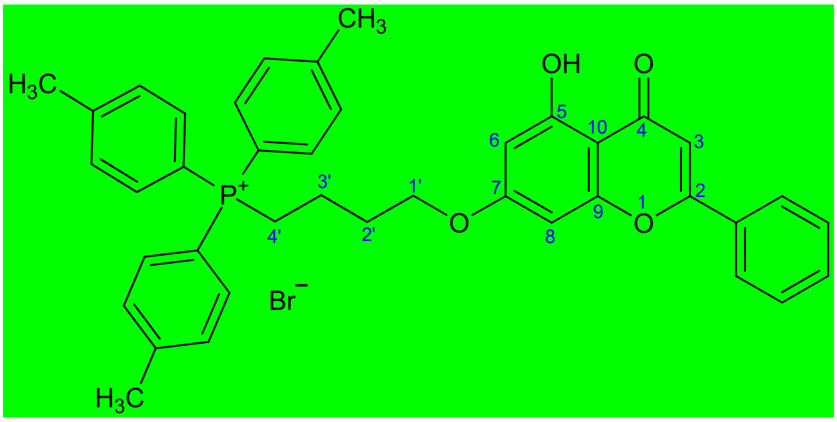

Figure S2. The skeleton numbering of compound **3b** used for NMR assignment.

S.1.2.3. Preparation of (4-((5-hydroxy-4-oxo-2-phenyl-4*H*-chromen-7-yl)oxy)butyl)tris(4-methoxyphenyl)phosphonium bromide (3c)

7-(4-Bromobutoxy)chrysin (**4**) (100 mg, 0.255 mmol) and tris(4-methoxyphenyl)phosphine (**1c**) (137 mg, 0.389 mmol, 1.5 eq.) were dissolved in anhydrous toluene (5 mL) and refluxed under an Ar atmosphere. After 10 hours, an additional 91 mg (0.257 mmol, 1.0 eq.) of phosphine **1c** and DMF (4.5 mL) were added to the reaction mixture. Stirring was continued for a total of 20 hours. The solvent was removed under reduced pressure using a rotary evaporator. The crude product was triturated with tert-butyl methyl ether, and the resulting solid was collected by filtration through a glass filter. The crude crystals were purified by preparative TLC (DCM:MeOH = 10:1). After purification, 96 mg (51%) of product (**3c**) was isolated. M.p.: 102–104 ◦C. TLC (DCM:MeOH = 5:1); R_f = 0.71. ^{1}H NMR (499.9 MHz; DMSO-d_6) δ (ppm) 1.63-1.75 (2H; m; H_2-3′); 1.94 (2H; qui; *J* = 6.5 Hz; H_2-2′); 3.44-3.53 (2H; m; H_2-4′); 3.88 (9H; s; 3×P-Ph-OC<u>H</u>$_3$); 4.19 (2H; t; *J* = 6.1 Hz; H_2-1′); 6.37 (1H; d; *J* = 2.2 Hz; H-6); 6.78 (1H; d; *J* = 2.2 Hz; H-8); 7.06 (1H; s; H-3); 7.25-

7.30 (6H; m; 6×P-Ph: H_{meta}); 7.58-7.71 (9H; m; 2×C(2)-Ph: H_{meta}, C(2)-Ph: H_{para}, 6×P-Ph: H_{orto}); 8.07-8.13 (2H; m; 2×C(2)-Ph: H_{orto}); 12.81 (1H; s; C(5)-O<u>H</u>). ^{13}C NMR (125.7 MHz; DMSO-d_6) δ (ppm) 18.2 (d; *J* = 3.4 Hz; C-3′); 20.5 (d; *J* = 53.3 Hz; C-4′); 28.7 (d; *J* = 17.1 Hz; C-2′); 55.8 (3×P-Ph-O<u>C</u>H_3); 66.9 (C-1′); 93.3 (C-8); 98.4 (C-6); 104.8 (C-10); 105.3 (C-3); 109.3 (d; *J* = 93.5 Hz; 3×P-Ph: C_{ipszo}); 115.8 (d; *J* = 13.6 Hz; 6×P-Ph: C_{meta}); 126.3 (2×C(2)-Ph: C_{orto}); 129.1 (2×C(2)-Ph: C_{meta}); 130.5 (C(2)-Ph: C_{ipszo}); 132.1 (C(2)-Ph: C_{para}); 135.4 (d; *J* = 11.5 Hz; 6×P-Ph: C_{orto}); 157.2 (C-9); 161.1 (C-5); 163.4 (C-2); 163.9 (d; *J* = 2.8 Hz; 3×P-Ph: C_{para}); 164.4 (C-7); 182.0 (C-4). HRMS: M^+=661.23387 (delta=-1.7 ppm; $C_{40}H_{38}O_7P$), HR-ESI-MS-MS (CID=60%; rel, int, %): 407(100); 379(56); 365(10); 352(29); 267(2); 245(4).

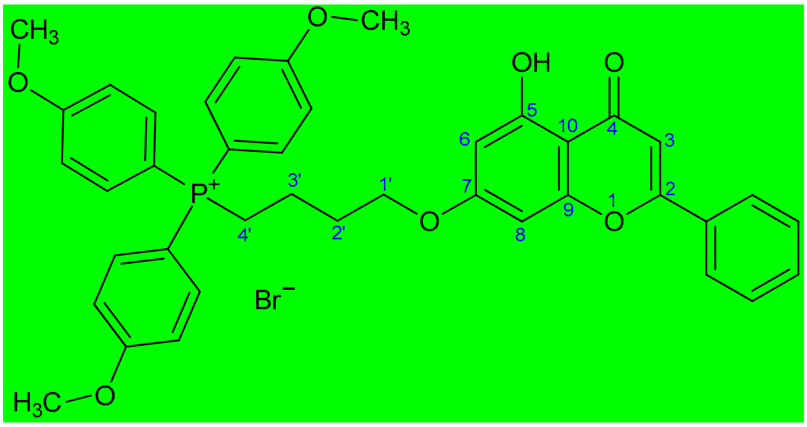

Figure S3. The skeleton numbering of compound **3c** used for NMR assignment.

S.1.2.4. Preparation of tris(4-fluorophenyl)(4-((5-hydroxy-4-oxo-2-phenyl-4*H*-chromen-7-yl)oxy)butyl)phosphonium bromide (3d)

7-(4-Bromobutoxy)chrysin (**4**) (100 mg, 0.257 mmol) and tris(4-fluorophenyl)phosphine (**1d**) (123 mg, 0.389 mmol, 1.5 eq.) were added to a round-bottom flask and dissolved in anhydrous toluene (5 mL). After 4 hours, an additional 205 mg (0.648 mmol, 2.5 eq.) of phosphine **1d** was added, followed later by a further amount (492 mg, 1.56 mmol, 6 eq.). To prevent precipitation, an additional 2 mL of anhydrous toluene and 0.5 mL of DMF were added to the reaction mixture. The solution was refluxed under an Ar atmosphere for 47 hours. After completion, the solvent was removed under reduced pressure. The resulting crude product was washed with tert-butyl methyl ether, and the solid was collected by filtration. Purification was carried out by preparative TLC (DCM:MeOH = 10:1), yielding the phosphonium salt **3d** as a solid (84 mg, 46%). M.p.: 64–66 °C. TLC (DCM:MeOH = 10:1); R_f = 0.47. ^{1}H NMR (499.9 MHz; DMSO-d_6) δ (ppm) 1.65-1.78 (2H; m; H_2-3′); 1.94 (2H; qui; *J* = 6.6 Hz; H_2-2′); 3.64-3.75 (2H; m; H_2-4′); 4.18 (2H; t; *J* = 6.2 Hz; H_2-1′); 6.36 (1H; d; *J* = 2.2 Hz; H-6); 6.76 (1H; d; *J* = 2.2 Hz; H-8); 7.06 (1H; s; H-3); 7.57-7.70 (9H; m; 2×C(2)-Ph: H_{meta}, C(2)-Ph: H_{para}, 6×P-PhF: H_{meta}); 7.88-7.98 (6H; m; 6×P-PhF: H_{orto}); 8.08-8.13 (2H; m; 2×C(2)-Ph: H_{orto}); 12.82 (1H; s; C(5)-O<u>H</u>). ^{13}C NMR (125.7 MHz; DMSO-d_6) δ (ppm) 18.0 (d; *J* = 3.5 Hz; C-3′); 20.0 (d; *J* = 50.8 Hz; C-4′); 28.7 (d; *J* = 17.4 Hz; C-2′); 66.9 (C-1′); 93.3 (C-8); 98.4 (C-6); 104.8 (C-10); 105.3 (C-3); 114.5 (dd; *J* = 90.4, 3.0 Hz; 3×P-PhF: C_{ipszo}); 117.8 (dd; *J* = 22.3, 14.0 Hz; 6×P-PhF: C_{meta}); 126.3 (2×C(2)-Ph: C_{orto}); 129.1 (2×C(2)-Ph: C_{meta}); 130.5 (C(2)-Ph: C_{ipszo}); 132.1 (C(2)-Ph: C_{para}); 136.9 (dd; *J* = 11.9, 10.0 Hz; 6×P-Ph: C_{orto}); 157.2 (C-9); 161.1 (C-5); 163.4 (C-2); 164.6 (C-7); 165.9 (dd; *J* = 255.9, 3.3 Hz; 3×P-PhF: C_{para}); 182.0 (C-4). HRMS: M^+=625.17496 (delta=-0.1 ppm; $C_{37}H_{29}O_4F_3P$), HR-ESI-MS-MS (CID=45%; rel, int, %): 371(100); 343(62); 329(11); 316(25); 309(9); 281(4); 267(30); 255(9); 235(5).

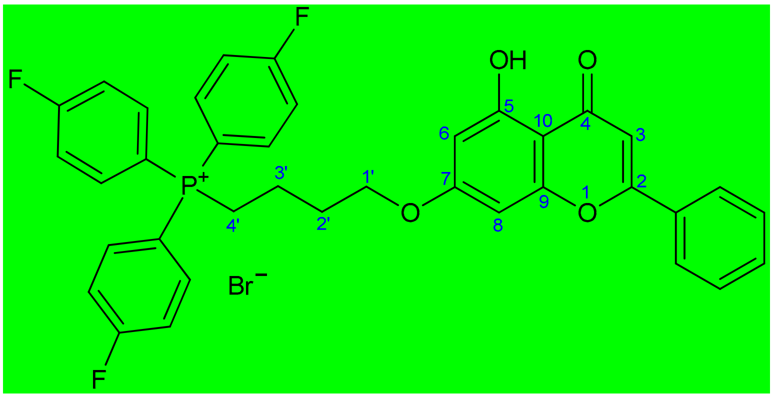

Figure S4. The skeleton numbering of compound **3d** used for NMR assignment.

S.1.2.5. Preparation of tricyclohexyl(4-((5-hydroxy-4-oxo-2-phenyl-4*H*-chromen-7-yl)oxy)butyl)phosphonium bromide (3e)

Anhydrous toluene (5 mL) was added to a pressure-resistant vessel, followed by the addition of 7-(4-bromobutoxy)chrysin (**4**) (100 mg, 0.257 mmol) and tricyclohexylphosphine (**1e**) (187 mg, 0.667 mmol, 2.6 eq.). The reaction mixture was initially heated to 115 °C, then the temperature was increased to 140 °C. The mixture was stirred under an Ar atmosphere for 59 hours. After completion, the mixture was evaporated under reduced pressure. The resulting crude product was triturated with tert-butyl methyl ether, and the solid was purified by preparative TLC (DCM:MeOH = 10:1). After purification, 95 mg (55%) of the target compound (**3e**) was isolated. M.p.: 76–78 ◦C. TLC (DCM:MeOH = 10:1); R_f = 0.40. ^{1}H NMR (499.9 MHz; DMSO-d_6) δ (ppm) 1.16-1.42 (9H; m; H_x-3a, H_x-3b, H_x-3c, H_x-4a, H_x-4b, H_x-4c, H_x-5a, H_x-5b, H_x-5c); 1.43-1.56 (6H; m; H_x-2a, H_x-2b, H_x-2c, H_x-6a, H_x-6b, H_x-6c); 1.59-1.84 (11H; m; H_2-3′, H_y-3a, H_y-3b, H_y-3c, H_y-4a, H_y-4b, H_y-4c, H_y-5a, H_y-5b, H_y-5c); 1.83-2.01 (8H; H-2′, H_y-2a, H_y-2b, H_y-2c, H_y-6a, H_y-6b, H_y-6c); 2.29-2.41 (2H; m; H-4′); 2.50-2.60 (3H; m; H-1a, H-1b, H-1c); 4.23 (2H; t; *J* = 6.0 Hz; H_2-1′); 6.42 (1H; s; H-6); 6.84 (1H; s; H-8); 7.06 (1H; s; H-3); 7.57-7.68 (3H; m; 2×C(2)-Ph: H_{meta}, C(2)-Ph: H_{para}); 8.08-8.13 (2H; m; 2×C(2)-Ph: H_{orto}); 12.82 (1H; br s; C(5)-O<u>H</u>). ^{13}C NMR (125.7 MHz; DMSO-d_6) δ (ppm) 13.5 (d; *J* = 43.3 Hz; C-4′); 18.0 (d; *J* = 4.5 Hz; C-3′); 25.7 (d; *J* = 3.9 Hz; C-4a, C-4b, C-4c); 25.8 (d; *J* = 12.1 Hz; C-3a, C-3b, C-3c, C-5a, C-5b, C-5c); 25.9 (d; *J* = 3.4 Hz; C-2a, C-2b, C-2c, C-6a, C-6b, C-6c); 28.4 (d; *J* = 41.2 Hz; C-1a, C-1b, C-1c); 29.1 (d; *J* = 14.7 Hz; C-2′); 66.8 (C-1′); 93.2 (C-8); 98.5 (C-6); 104.9 (C-10); 105.3 (C-3); 126.3 (2×C(2)-Ph: C_{orto}); 129.1 (2×C(2)-Ph: C_{meta}); 130.5 (C(2)-Ph: C_{ipszo}); 132.1 (C(2)-Ph: C_{para}); 157.3 (C-9); 161.1 (C-5); 163.4 (C-2); 164.4 (C-7); 181.9 (C-4). HRMS: M^+=589.34368 (delta=-0.8 ppm; $C_{37}H_{50}O_4P$), HR-ESI-MS-MS (CID=45%; rel, int, %): 507(36); 335(17); 253(100); 171(3).

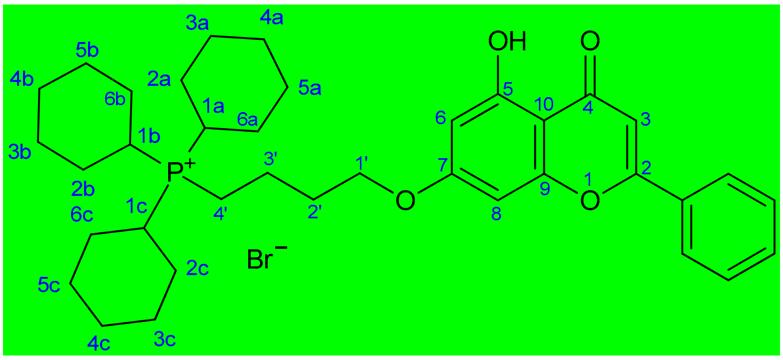

Figure S5. The skeleton numbering of compound **3e** used for NMR assignment.

S.1.2.6. Preparation of (4-((5-hydroxy-4-oxo-2-phenyl-4*H*-chromen-7-yl)oxy)butyl)tris(4-methoxy-3,5-dimethylphenyl)phosphonium bromide (3f)

7-(4-Bromobutoxy)chrysin (**4**) (60 mg, 0.154 mmol) and tris(4-methoxy-3,5-dimethylphenyl)phosphine (**1f**) (202 mg, 0.462 mmol, 3 equiv.) were dissolved in anhydrous acetonitrile (5 mL) with a few drops of DMF in a pressure-resistant vessel. The reaction mixture was stirred under an Ar atmosphere at 115 °C for 12 hours. After completion, the precipitated crystals were filtered off, and the remaining solution was evaporated under reduced pressure. The crude product was purified by preparative TLC (DCM:MeOH = 10:1), affording the desired compound **3f** as a solid (99 mg, 78%). M.p.: 93–95 ◦C. TLC (DCM:MeOH = 10:1); R_f = 0.52. ^{1}H NMR (599.8 MHz; DMSO-d_6) δ (ppm) 1.58-1.71 (2H; m; H_2-3′); 1.93 (2H; qui; *J* = 6.1 Hz; H_2-2′); 2.28 (18H; s; 6×P-Ar: C<u>H₃</u>); 3.42-3.51 (2H; m; H_2-4′); 3.76 (9H; s; 3×P-Ar: OC<u>H</u>$_3$); 4.20 (2H; t; *J* = 5.8 Hz; H_2-1′); 6.34 (1H; d; *J* = 1.1 Hz; H-6); 6.75 (1H; d; *J* = 1.1 Hz; H-8); 7.06 (1H; s; H-3); 7.50 (6H; d; *J* = 12.5 Hz; 6× P-Ar: H_{orto}); 7.57-7.68 (3H; m; 2×C(2)-Ph: H_{meta}, C(2)-Ph: H_{para}); 8.10 (2H; br d; *J* = 7.5 Hz; 2×C(2)-Ph: H_{orto}); 12.81 (1H; s; C(5)-O<u>H</u>). ^{13}C NMR (150.8 MHz; DMSO-d_6) δ (ppm) 16.0 (6×P-Ar: <u>C</u>H$_3$); 18.1 (d; *J* = 3.4 Hz; C-3′); 20.0 (d; *J* = 53.3 Hz; C-4′); 28.7 (d; *J* = 17.1 Hz; C-2′); 59.6 (3×P-Ar: O<u>C</u>H$_3$); 66.9 (C-1′); 93.3 (C-8); 98.4 (C-6); 104.9 (C-10); 105.4 (C-3); 113.4 (d; *J* = 88.0 Hz; 3×P-Ar: C_{ipszo}); 126.4 (2×C(2)-Ph: C_{orto}); 129.2 (2×C(2)-Ph: C_{meta}); 130.6 (C(2)-Ph: C_{ipszo}); 132.2 (C(2)-Ph: C_{para}); 133.1 (d; *J* = 14.8 Hz; 6×P-Ar: C_{meta}); 133.8 (d; *J* = 10.6 Hz; 6×P-Ar: C_{orto}); 157.3 (C-9); 161.2 (C-5); 161.7 (d; *J* = 3.5 Hz; 3×P-Ar: C_{para}); 163.5 (C-2); 164.5 (C-7); 182.0 (C-4). HRMS: M^+=745.32812 (delta=-1.0 ppm; $C_{46}H_{50}O_7P$), HR-ESI-MS-MS (CID=45%; rel, int, %): 730(100); 715(22); 701(24); 491(52); 475(13); 463(27); 447(48); 436(14); 420(23).

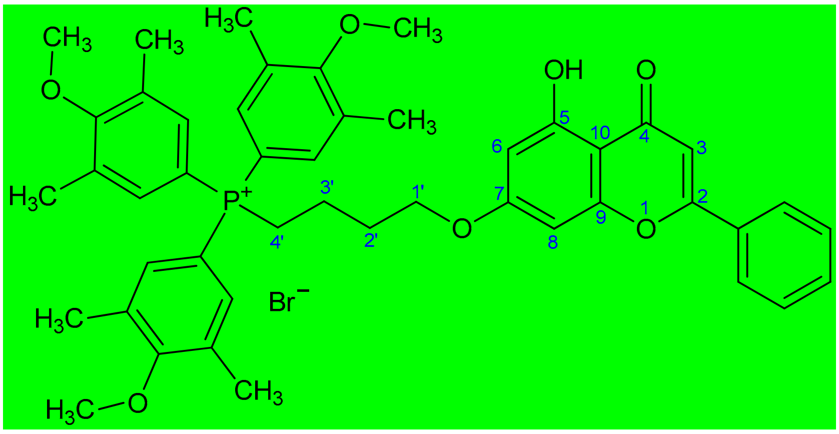

Figure S6. The skeleton numbering of compound **3f** used for NMR assignment.

S.1.2.7. Preparation of (4-((5-hydroxy-4-oxo-2-phenyl-4*H*-chromen-7-yl)oxy)butyl)tri-o-tolylphosphonium bromide (3g)

7-(4-Bromobutoxy)chrysin (**4**) (100 mg, 0.257 mmol) and tri-*o*-tolylphosphine (**1g**) (312 mg, 1.025 mmol, 4 eq.) were dissolved in anhydrous acetonitrile (10 mL) in a pressure-resistant vessel. The reaction mixture was stirred at 105 °C under an Ar atmosphere. After 10 hours, an additional 156 mg (0.514 mmol, 2 eq.) of phosphine **1g** was added along with a few drops of DMF to aid dissolution. Following a total reaction time of 45 hours, the mixture was evaporated under reduced pressure. The precipitated solid was washed with tert-butyl methyl ether. The crude product was purified by preparative TLC (DCM:MeOH = 10:1), yielding the phosphonium salt **3g** as a solid (25 mg, 14%). M.p.: 79–81 ◦C. TLC (DCM:MeOH = 10:1); R_f = 0.38. ^{1}H NMR (599.8 MHz; DMSO-d_6) δ (ppm) 1.50 (2H; br s; H_2-3′); 2.05 (2H; qui; *J* = 6.4 Hz; H_2-2′); 2.18 (9H; s; 3×C(2″)-C<u>H</u>$_3$); 3.67 (2H; ~td; *J* = 10.3, 6.0 Hz; H_2-4′); 4.19 (2H; t; *J* = 5.9 Hz; H_2-1′); 6.36 (1H; d; *J* = 1.2 Hz; H-6); 6.78 (1H; d; *J* = 1.2 Hz; H-8); 7.06 (1H; s; H-3); 7.50-7.68 (12H; m; 2×C(2)-Ph: H_{meta}, C(2)-Ph: H_{para}, 3×H-3″, 3×H-5″, 3×H-6″); 7.82 (3H; br t; *J* = 6.8 Hz; 3×H-4″); 8.10 (2H; br d; *J* = 7.5 Hz; 2×C(2)-Ph: H_{orto}); 12.81 (1H; s; C(5)-O<u>H</u>). ^{13}C NMR (150.8 MHz; DMSO-d_6) δ (ppm) 21.2 (d; *J* = 3.4 Hz; C-3′); 21.9 (d; *J* = 52.2 Hz; C-4′); 22.5 (d; *J* = 4.2 Hz; 3×C(2″)-<u>C</u>H$_3$); 28.9 (d; *J* = 16.9 Hz; C-2′); 67.3 (C-1′); 93.8 (C-8); 98.9 (C-6); 105.5 (C-10); 105.9 (C-3); 116.7 (d; *J* = 88.3 Hz; 3×C-1″); 126.9 (2×C(2)-Ph: C_{orto}); 128.2 (d; *J* = 12.7; 3×C-5″); 129.6 (2×C(2)-Ph: C_{meta}); 131.0 (C(2)-Ph: C_{ipszo}); 132.7 (C(2)-Ph: C_{para}); 134.0 (d; *J* = 11.6 Hz; 3×C-3″); 135.0 (d; *J* = 11.7 Hz; 3×C-6″); 135.6 (br s; 3×C-4″); 143.7 (d; *J* = 8.5 Hz; 3×C-2″); 157.8 (C-9); 161.6 (C-5); 163.4 (C-2); 164.9 (C-7); 182.5 (C-4). HRMS: M^+=613.25021 (delta=-0.02 ppm; $C_{40}H_{38}O_4P$), HR-ESI-MS-MS (CID=45%; rel, int, %): 359(100); 345(47); 309(23); 303(18); 289(71); 267(63); 255(26); 237(10).

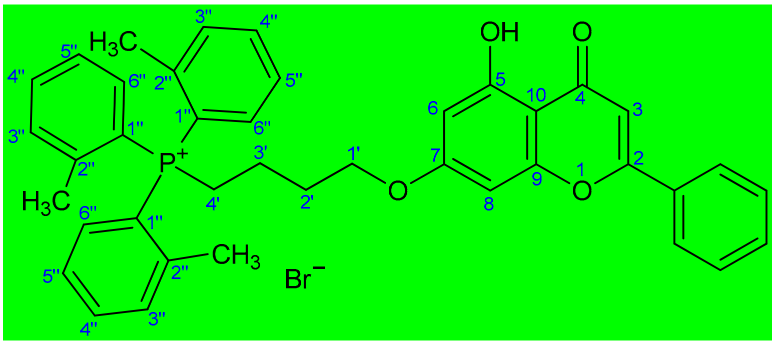

Figure S7. The skeleton numbering of compound **3g** used for NMR assignment.

S.1.2.8. Preparation of (4-((5-hydroxy-4-oxo-2-phenyl-4*H*-chromen-7-yl)oxy)butyl)diphenyl(pyridin-2-yl)phosphonium bromide (3h)

7-(4-Bromobutoxy)chrysin (**4**) (100 mg, 0.257 mmol) and diphenyl-2-pyridylphosphine (**1h**) (203 mg, 0.771 mmol, 3 eq.) were weighed into a pressure-resistant vessel and dissolved in anhydrous toluene (5 mL). The reaction mixture was stirred at 115 °C under an Ar atmosphere. After 5 hours, an additional 68 mg (0.257 mmol, 1 eq.) of phosphine **1h** was added. After 15 hours, the reaction mixture was processed due to the increasing amount of a side product. The precipitated crystals were filtered and purified by preparative TLC (DCM:MeOH = 15:1). After purification, 45 mg (27%) of product (**3h**) was isolated. M.p.: 82–84 ◦C. TLC (DCM:MeOH = 12:1); R_f = 0.38. ^{1}H NMR (599.8 MHz; DMSO-d_6) δ (ppm) 1.73-1.83 (2H; m; H_2-3′); 1.96 (2H; qui; *J* = 7.0 Hz; H_2-2′); 3.64-3.74 (2H; m; H_2-4′); 4.17 (2H; t; *J* = 6.5 Hz; H_2-1′); 6.34 (1H; d; *J* = 2.4 Hz; H-6); 6.77 (1H; d; *J* = 2.2 Hz; H-8); 7.05 (1H; s; H-3); 7.56-7.66 (3H; m; 2×C(2)-

Ph: H_{meta}, C(2)-Ph: H_{para}); 7.73-7.79 (4H; m; 4×P-Ph: H_{meta}); 7.82-7.92 (7H; m; H-4″, 4×P-Ph: H_{orto}, 2×P-Ph: H_{para}); 7.96-8.00 (1H; m; H-6″); 8.08-8.12 (2H; m; 2×C(2)-Ph: H_{orto}); 8.19 (1H; tdd; *J* = 7.81, 5.2, 1.7 Hz; H-5″); 9.00 (1H; br d; *J* = 4.7 Hz; H-3″); 12.80 (1H; s; C(5)-O<u>H</u>). ^{13}C NMR (150.8 MHz; DMSO-d_6) δ (ppm) 18.4 (d; *J* = 4.5 Hz; C-3′); 19.4 (d; *J* = 50.2 Hz; C-4′); 28.9 (d; *J* = 16.7 Hz; C-2′); 67.2 (C-1′); 93.3 (C-8); 98.5 (C-6); 104.9 (C-10); 105.4 (C-3); 117.7 (d; *J* = 85.8 Hz; 2×P-Ph: C_{ipszo}); 126.4 (2×C(2)-Ph: C_{orto}); 128.4 (C-4″); 129.2 (2×C(2)-Ph: C_{meta}); 130.1 (d; *J* = 12.5 Hz; 4×P-Ph: C_{meta}); 130.6 (C(2)-Ph: C_{ipszo}); 131.3 (d; *J* = 23.3 Hz; C-6″); 132.2 (C(2)-Ph: C_{para}); 133.8 (d; *J* = 10.6 Hz; 4×P-Ph: C_{orto}); 135.0 (d; *J* = 2.9 Hz; 2×P-Ph: C_{para}); 138.3 (d; *J* = 10.1 Hz; C-5″); 144.4 (d; *J* = 116.0 Hz; C-1″); 152.1 (d; *J* = 19.1 Hz; C-3″); 157.3 (C-9); 161.1 (C-5); 163.5 (C-2); 164.5 (C-7); 182.0 (C-4). HRMS: M^+=572.19641 (delta=-3.7 ppm; $C_{36}H_{31}O_4NP$), HR-ESI-MS-MS (CID=45%; rel, int, %): 318(100); 309(13); 290(41); 267(34); 262(20); 255(13); 186(10).

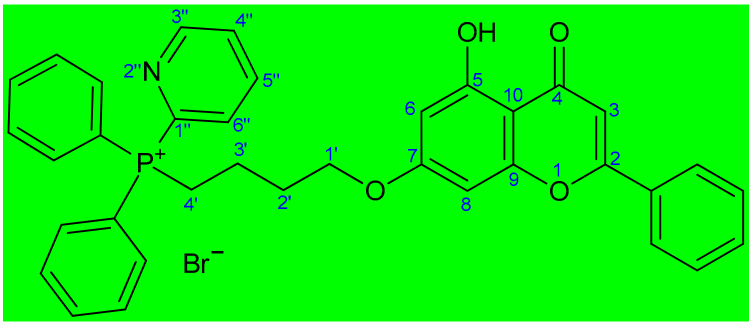

Figure S8. The skeleton numbering of compound **3h** used for NMR assignment.

S.1.2.9. Preparation of (4-((5-hydroxy-4-oxo-2-phenyl-4*H*-chromen-7-yl)oxy)butyl)(2-methoxyphenyl)diphenylphosphonium bromide (3i)

7-(4-Bromobutoxy)chrysin (**4**) (100 mg, 0.257 mmol) and diphenyl(2-methoxyphenyl)phosphine (**1i**) (229 mg, 0.771 mmol, 3 eq.) were dissolved in anhydrous acetonitrile (5 mL) in a pressure-resistant vessel. The reaction mixture was stirred under an Ar atmosphere for 19 hours. After completion, the solvent was removed under reduced pressure. The resulting crude product was triturated with tert-butyl methyl ether. Finally, the product was purified by preparative TLC (DCM:MeOH = 10:1), yielding the desired compound **3i** as a solid (156 mg, 89%). M.p.: 71–72 °C. TLC (DCM:MeOH = 10:1); R_f = 0.52. ^{1}H NMR (599.8 MHz; DMSO-d_6) δ (ppm) 1.67 (2H; ~hex; *J* = 7.3 Hz; H_2-3′); 1.96 (2H; quin; *J* = 6.5 Hz; H_2-2′); 3.50-3.61 (2H; m; H_2-4′); 3.70 (3H; s; C(2″)-OC<u>H</u>$_3$); 4.19 (2H; t; *J* = 6.1 Hz; H_2-1′); 6.35 (1H; d; *J* = 1.7 Hz; H-6); 6.78 (1H; d; *J* = 1.7 Hz; H-8); 7.06 (1H; s; H-3); 7.31 (1H; td; *J* = 7.6, 2.3 Hz; H-5″); 7.38-7.46 (2H; m; H-3″, H-6″); 7.57-7.67 (3H; m; 2×C(2)-Ph: H_{meta}, C(2)-Ph: H_{para}); 7.70-7.80 (8H; m; 4×P-Ph: H_{orto}, 4×P-Ph: H_{meta}); 7.84-7.89 (2H; m; 2×P-Ph: H_{para}); 7.92 (1H; t; *J* = 7.9 Hz; H-4″); 8.08-8.12 (2H; m; 2×C(2)-Ph: H_{orto}); 12.80 (1H; s; C(5)-O<u>H</u>). ^{13}C NMR (150.8 MHz; DMSO-d_6) δ (ppm) 19.0 (d; *J* = 4.2 Hz; C-3′); 20.8 (d; *J* = 51.7 Hz; C-4′); 28.9 (d; *J* = 17.5 Hz; C-2′); 56.3 (C(2″)-O<u>C</u>H$_3$); 67.1 (C-1′); 93.3 (C-8); 98.5 (C-6); 104.9 (C-10); 105.3 (d; *J* = 88.8 Hz; C-1″); 105.4 (C-3); 113.4 (d; *J* = 6.4 Hz; C-3″); 118.8 (d; *J* = 88.0 Hz; 2×P-Ph: C_{ipszo}); 122.3 (d; *J* = 12.7 Hz; C-5″); 126.4 (2×C(2)-Ph: C_{orto}); 129.2 (2×C(2)-Ph: C_{meta}); 130.0 (d; *J* = 12.5 Hz; 4×P-Ph: C_{meta}); 130.6 (C(2)-Ph: C_{ipszo}); 132.2 (C(2)-Ph: C_{para}); 133.0 (d; *J* = 10.3 Hz; 4×P-Ph: C_{orto}); 134.4 (d; *J* = 2.9 Hz; 2×P-Ph: C_{para}); 135.0 (d; *J* = 8.5 Hz; C-6″); 137.9 (C-4″); 157.3 (C-9); 161.1 (C-5); 161.8 (d; *J* = 1.9 Hz; C-2″); 163.5 (C-2); 164.5 (C-7); 182.0 (C-4). HRMS: M^+=601.21250 (delta=-2.2 ppm; $C_{38}H_{34}O_5P$), HR-ESI-MS-MS (CID=45%; rel, int, %): 347(100); 333(15); 319(37); 291(9); 277(16); 267(14); 255(5); 199(4).

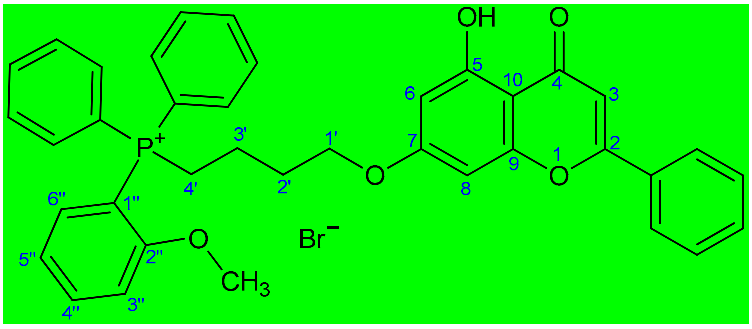

Figure S9. The skeleton numbering of compound **3i** used for NMR assignment.

S.1.2.10. Preparation of *tert*-butyl(4-((5-hydroxy-4-oxo-2-phenyl-4*H*-chromen-7-yl)oxy)butyl)diphenylphosphonium bromide (3j)

7-(4-Bromobutoxy)chrysin (**4**) (80 mg, 0.206 mmol) and *tert*-butyldiphenylphosphine (**1j**) (149 mg, 0.617 mmol, 3 eq.) were weighed into a pressure-resistant vessel and dissolved in anhydrous acetonitrile (5 mL). The reaction mixture was stirred at 110 °C under an Ar atmosphere for 18 hours. After completion, the mixture was evaporated under reduced pressure. The crude product was purified by preparative TLC (DCM:MeOH = 8:1), affording the desired product **3j** as a solid (77 mg, 59%). M.p.: 86–88 °C. TLC (DCM:MeOH = 8:1); R_f = 0.56. ^{1}H NMR (599.8 MHz; DMSO-d_6) δ (ppm) 1.37 (9H; d; *J* = 16.7 Hz; 3×P-tBu: C<u>H</u>$_3$); 1.46 (2H; ~hex; *J* = 7.7 Hz; H$_2$-3′); 1.93 (2H; quin; *J* = 7.0 Hz; H$_2$-2′); 3.23-3.30 (2H; m; H$_2$-4′); 4.14 (2H; t; *J* = 6.4 Hz; H$_2$-1′); 6.32 (1H; d; *J* = 2.2 Hz; H-6); 6.75 (1H; d; *J* = 2.2 Hz; H-8); 7.04 (1H; s; H-3); 7.57-7.66 (3H; m; 2×C(2)-Ph: H_{meta}, C(2)-Ph: H_{para}); 7.73-7.80 (4H; m; 4×P-Ph: H_{meta}); 7.87-7.92 (2H; m; 2×P-Ph: H_{para}); 7.93-7.99 (4H; m; 4×P-Ph: H_{orto}); 8.07-8.12 (2H; m; 2×C(2)-Ph: H_{orto}); 12.81 (1H; s; C(5)-O<u>H</u>). ^{13}C NMR (150.8 MHz; DMSO-d_6) δ (ppm) 15.9 (d; *J* = 46.6 Hz; C-4′); 18.7 (d; *J* = 5.3 Hz; C-3′); 25.3 (3×P-tBu: <u>C</u>H$_3$); 28.7 (d; *J* = 15.9 Hz; C-2′); 32.6 (d; *J* = 42.9 Hz; P-tBu: <u>C</u>(CH$_3$)$_3$); 67.1 (C-1′); 93.2 (C-8); 98.4 (C-6); 104.8 (C-10); 105.3 (C-3); 116.0 (d; *J* = 77.1 Hz; 2×P-Ph: C_{ipszo}); 126.3 (2×C(2)-Ph: C_{orto}); 129.1 (2×C(2)-Ph: C_{meta}); 129.9 (d; *J* = 11.4 Hz; 4×P-Ph: C_{meta}); 130.5 (C(2)-Ph: C_{ipszo}); 132.1 (C(2)-Ph: C_{para}); 134.1 (d; *J* = 8.5 Hz; 4×P-Ph: C_{orto}); 134.5 (d; *J* = 2.9 Hz; 2×P-Ph: C_{para}); 157.2 (C-9); 161.0 (C-5); 163.4 (C-2); 164.4 (C-7); 181.9 (C-4). HRMS: M^+=551.23604 (delta=2.7 ppm; $C_{35}H_{36}O_4P$), HR-ESI-MS-MS (CID=45%; rel, int, %): 495(97); 241(100).

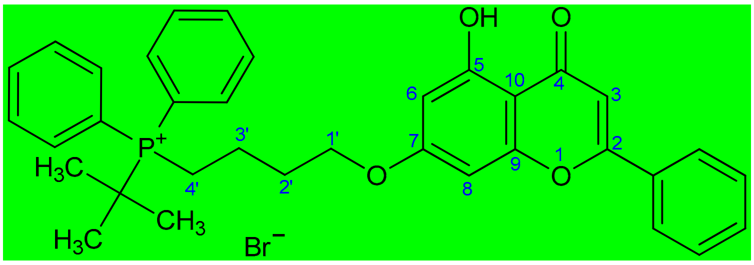

Figure S10. The skeleton numbering of compound **3j** used for NMR assignment.

S.1.2. Preparation of 5-hydroxy-4-oxo-2-phenyl-4*H*-chromen-7-yl 3,3,3-triphenylpropanoate (5)

3,3,3-Triphenylpropanoic acid (**2**) (302 mg, 1.00 mmol, 1.4 eq.) was dissolved in 8 mL of anhydrous DCM in a round-bottom flask, followed by the addition 1 mL (13.8 mmol, 19.4 eq.) of thionyl chloride. The reaction mixture was stirred at 60 °C for 5 hours. After completion, the solution was evaporated to dryness, and the crude acid chloride was used without further purification. Solution A was prepared by dissolving the crude acid chloride in 5 mL of acetone. Solution B was prepared by dissolving 184 mg (0.712 mmol) of chrysin (**2**) in 5 mL of acetone, followed by the addition of 0.24 mL (1.71 mmol, 2.4 eq.) of triethylamine. At 0 °C, solution A was added dropwise to solution B under an Ar atmosphere, and the reaction mixture was stirred at 45 °C for 6 hours. After completion, the resulting solid was filtered off, and the solution was evaporated. The crude product was dissolved in 10 mL of DCM and washed with 7 mL of water, followed by stirring for 10 minutes. The mixture was extracted with DCM (3 * 15 mL), the combined organic layers were dried over $MgSO_4$, then the solution was filtered and evaporated. The crude material was purified by preparative TLC (DCM:MeOH = 20:1). Ultimately, the desired product **5** was obtained as a solid (194 mg, 60%). M.p.: 172–174 °C. TLC (Heptane:EtOAc = 3:2); R_f = 0.57. ^{1}H NMR (599.8 MHz; DMSO-d_6) δ (ppm) 4.14 (3H; s; H$_2$-2′); 6.08 (1H; d; *J* = 1.8 Hz; H-6); 6.61 (1H; d; *J* = 1.8 Hz; H-8); 7.12 (1H; s; H-3);7.22-7.28 (3H; m; 3×C(3′)-Ph: H_{para}); 7.28-7.37 (12H; m; 6×C(3′)-Ph: H_{orto}, 6× C(3′)-Ph: H_{meta}); 7.57-7.67 (3H; m; 2×C(2)-Ph: H_{meta}, C(2)-Ph: H_{para}); 8.07-8.13 (2H; m; 2×C(2)-Ph: H_{orto}); 12.77 (1H; s; C(5)-O<u>H</u>). ^{13}C NMR (150.8 MHz; DMSO-d_6) δ (ppm) 45.2 (C-2′); 55.5 (C-3′); 101.1 (C-8); 104.8 (C-6); 105.6 (C-3); 108.0 (C-10); 126.1 (3×C(3′)-Ph: C_{para}); 126.5 (2×C(2)-Ph: C_{orto}); 127.8 (6×C(3′)-Ph: C_{meta}); 128.8 (6×C(3′)-Ph: C_{orto}); 129.1 (2×C(2)-Ph: C_{meta}); 130.2 (C(2)-Ph: C_{ipszo}); 132.3 (C(2)-Ph: C_{para}); 146.0 (3×C(3′)-Ph: C_{ipszo}); 155.2 (C-7); 156.0 (C-9); 160.5 (C-5); 164.1 (C-2); 168.2 (C-1′); 182.4 (C-4). HRMS: M+H=539.18440 (delta=-1.7 ppm; $C_{36}H_{27}O_5$), HR-ESI-MS-MS (CID=45%; rel, int, %): 255(100).

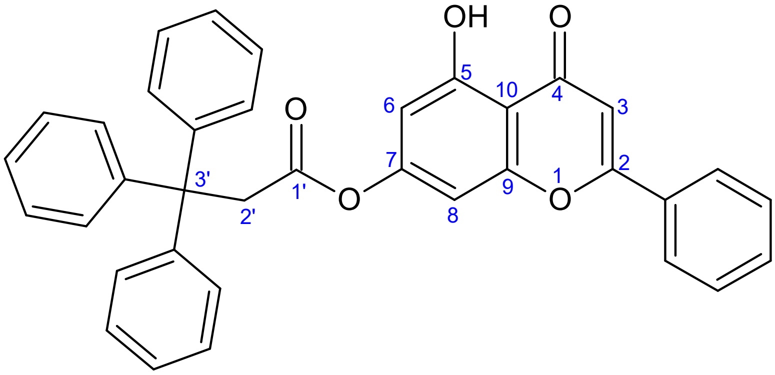

Figure S11. The skeleton numbering of compound **5** used for NMR assignment.

S.2. NCI60 Screening Results

Table S1. Growth percent rates of chrysin (**2**), and compounds **3a–h** at the concentration of 10 µM against 60 human cancer cell lines in vitro. All data with greater than 70% loss of cells are highlighted in bold. n.d. means not determined.

	2	3a	3b	3c	3d	3e	3f	3g	3h
Leukemia									
CCRF-CEM	102.24	**−75.89**	**−70.45**	**−71.68**	−55.86	−66.74	−32.62	**−96.55**	−20.73
HL-60(TB)	116.20	−51.48	−60.44	−31.16	−46.93	−20.64	−27.66	**−97.42**	0.68
K-562	96.80	−24.12	−4.21	−39.75	−17.57	3.17	6.85	**−97.50**	−51.29
MOLT-4	105.93	−41.74	−43.39	−51.58	−24.30	−60.61	−42.47	**−98.60**	21.06
RPMI-8226	101.04	−66.65	−55.76	−67.50	−30.06	−62.59	−42.87	**−98.51**	−35.31
SR	77.87	−39.83	−36.14	n.d.	−36.14	n.d.	23.00	n.d.	0.35
Non-small Cell Lung Cancer									
A549/ATCC	98.46	−56.00	−49.46	**−91.17**	−41.14	**−95.33**	−66.07	**−94.13**	−43.45
EKVX	89.35	**−97.60**	**−88.30**	**−70.86**	**−83.91**	**−83.52**	**−76.52**	**−98.39**	**−93.14**
HOP-62	113.09	−63.56	−61.95	**−81.09**	**−70.75**	**−82.57**	−25.25	**−98.29**	−56.95
HOP-92	77.93	n.d.	n.d.	**−79.86**	n.d.	**−75.86**	−68.75	**−98.85**	−69.74
NCI-H226	86.84	−29.00	−50.16	−67.61	−56.94	−56.69	−58.48	**−99.39**	−50.43
NCI-H23	92.57	**−93.04**	**−90.93**	**−77.05**	**−93.04**	**−88.52**	**−77.35**	**−98.38**	**−89.29**
NCI-H322M	98.30	**−100.00**	**−79.50**	−45.10	**−88.26**	**−85.23**	−62.17	**−98.16**	**−94.46**
NCI-H460	98.34	**−75.82**	−47.76	2.18	−47.38	−66.03	−58.60	**−91.49**	**−84.23**
NCI-H522	88.95	**−81.94**	**−74.44**	**−84.90**	**−74.44**	**−87.96**	**−77.26**	**−98.47**	**−72.26**
Colon Cancer									
COLO 205	104.94	−56.27	−64.54	−68.30	−68.44	**−70.25**	−62.18	**−96.97**	**−75.86**
HCC-2998	102.88	**−93.06**	**−88.67**	**−72.87**	**−88.99**	**−84.68**	**−73.48**	**−98.20**	**−88.50**
HCT-116	82.69	−58.14	1.29	**−95.49**	−66.56	−62.02	−47.60	**−94.45**	−61.65
HCT-15	80.99	**−94.23**	**−88.01**	−64.23	−48.58	**−88.85**	−58.86	**−94.66**	**−87.97**
HT29	102.89	−64.96	−60.66	**−82.78**	−69.75	**−84.91**	**−72.72**	**−97.86**	−60.61
KM12	92.93	**−85.22**	**−75.51**	**−86.20**	**−81.63**	**−92.05**	**−82.34**	**−95.80**	**−94.63**
SW-620	101.60	**−81.69**	−68.54	−34.84	−67.49	**−76.43**	−62.43	**−96.38**	**−88.28**
CNS Cancer									
SF-268	101.55	−58.96	−61.27	−38.32	**−71.57**	−67.46	−66.45	**−97.87**	−67.58
SF-295	99.86	**−96.05**	**−91.31**	**−73.77**	**−73.97**	**−87.04**	−63.61	**−98.68**	**−92.71**
SF-539	92.17	**−95.73**	**−80.55**	−66.86	**−89.58**	87.07	**−89.21**	**−97.67**	**−96.45**
SNB-19	86.04	−44.47	**−96.12**	**−78.93**	**−97.41**	**−87.60**	**−76.79**	**−97.41**	**−91.56**
SNB-75	88.98	**−80.72**	**−78.39**	−51.13	**−78.02**	**−80.53**	**−70.97**	**−98.99**	−47.32
U251	80.67	−57.37	−49.95	**−92.63**	−40.68	**−95.45**	**−76.62**	**−96.95**	**−96.00**
Melanoma									
LOX IMVI	85.08	**−90.07**	**−91.23**	**−78.83**	**−93.71**	**−90.52**	−68.69	**−97.49**	**−84.08**
MALME-3M	101.76	**−88.05**	−57.87	−45.01	**−82.37**	**−76.92**	−42.54	**−98.50**	**−93.37**
M14	106.78	**−70.11**	**−75.22**	−1.24	**−75.46**	**−82.15**	−68.31	**−97.53**	**−84.31**
MDA-MB-435	99.53	−67.47	**−88.23**	**−89.63**	**−90.81**	**−93.62**	**−81.87**	**−98.27**	**−80.81**
SK-MEL-2	109.90	**−94.08**	**−76.88**	**−88.56**	**−77.26**	**−91.86**	**−77.64**	**−98.45**	**−95.41**
SK-MEL-28	101.70	**−98.45**	**−85.28**	−44.48	**−92.00**	−41.35	**−85.74**	**−98.42**	**−94.65**
SK-MEL-5	92.85	**−98.40**	**−96.48**	**−93.91**	**−99.15**	**−100.00**	**−75.18**	**−97.86**	**−96.32**
UACC-257	118.94	**−91.03**	**−72.15**	**−94.74**	**−82.13**	**−92.15**	**−79.73**	**−99.31**	**−89.82**
UACC-62	82.24	−59.51	**−88.32**	n.d.	**−96.92**	n.d.	**−80.44**	**−97.94**	**−73.54**
Ovarian Cancer									
IGROV1	95.22	**−75.73**	−46.94	−6.99	−64.01	−59.44	−38.04	**−99.12**	**−80.13**
OVCAR-3	97.60	**−93.95**	**−88.23**	−68.07	**−71.99**	**−81.93**	−69.83	**−99.11**	**−93.67**
OVCAR-4	112.07	−69.14	**−73.12**	**−74.27**	−48.14	−64.45	−65.45	**−98.70**	−51.22
OVCAR-5	99.07	**−97.68**	**−80.28**	−59.27	**−89.87**	−60.46	**−87.76**	n.d.	**−93.39**
OVCAR-8	95.19	−61.09	−30.10	**−90.58**	−45.64	**−86.43**	−68.14	**−95.94**	**−91.64**
NCI/ADR-RES	92.84	−68.29	−65.69	−13.25	64.96	−20.84	−66.83	**−97.87**	−41.34
SK-OV-3	128.15	**−83.40**	**−75.29**	**−77.68**	**−88.20**	**−82.42**	−58.30	**−97.86**	**−76.55**
Renal Cancer									
786-0	99.20	−69.49	−69.80	−65.30	−56.53	**−80.09**	−47.00	**−98.09**	**−74.52**
A498	86.62	**−83.28**	**−81.52**	**−83.03**	**−87.56**	**−87.64**	**−83.41**	**−99.08**	**−94.03**
ACHN	85.03	**−100.00**	**−92.79**	−63.47	**−85.38**	**−72.49**	**−88.43**	**−96.98**	**−97.99**
CAKI-1	83.56	**−97.07**	**−90.39**	**−75.47**	**−93.64**	**−90.81**	−51.60	**−97.20**	**−71.23**
RXF 393	91.19	n.d.	**−74.82**	**−74.44**	**−83.98**	**−80.02**	−66.86	**−98.65**	**−80.52**
SN12C	85.77	−49.55	**−84.43**	−58.96	**−82.12**	−57.04	**−77.42**	**−97.05**	**−91.41**
TK-10	107.21	**−81.80**	**−79.34**	**−87.07**	**−84.81**	**−91.34**	**−84.67**	**−99.33**	**−93.27**
UO-31	89.42	**−97.08**	**−82.52**	**−89.89**	**−86.94**	**−92.53**	−59.61	n.d.	**−93.98**
Prostate Cancer									
PC-3	93.17	**−87.34**	n.d.	**−82.70**	n.d.	**−70.81**	**−76.48**	**−97.92**	−35.64
DU-145	92.00	**−94.82**	**−85.93**	**−90.69**	**−79.20**	**−96.10**	**−92.74**	**−97.10**	**−95.29**
Breast Cancer									
MCF7	103.05	**−79.07**	**−74.84**	−56.76	**−84.78**	**−77.03**	−57.96	**−95.41**	−62.56
MDA-MB-231/ATCC	82.64	−32.25	**−90.69**	**−72.06**	**−94.98**	**−81.62**	**−74.76**	n.d.	**−94.33**
HS 578T	92.51	−35.23	−52.21	−62.66	−54.51	−62.09	−57.66	**−98.33**	−35.75
BT-549	91.01	**−83.01**	−19.04	**−82.58**	**−75.95**	**−89.91**	−53.37	**−98.08**	−69.67
T-47D	101.44	−62.52	−52.52	n.d.	−53.27	n.d.	−52.77	n.d.	−49.47
MDA-MB-468	91.09	**−84.94**	**−87.04**	**−77.39**	**−85.72**	**−83.55**	**−75.97**	**−99.41**	**−90.71**
Mean									
	96.10	**−74.27**	−69.90	−68.29	−69.51	**−75.98**	−63.40	**−97.65**	**−72.32**

S.3. NCI60 Screening

A detailed description of the NCI screening procedures [36-38] can also be found on the website of NCI [39], and in our previous work [26].

S.3.1. One-Dose Screen

All candidates were examined at first at a single high dose (10 μ*M*) in the full NCI60 cell panel [36-39]. The value reported for the one-dose test is growth relative to the no-drug control and relative to the time zero number of cells. This made possible the disclosure of both growth inhibition (numbers between 0 and 100) and lethality (numbers less than 0). For example, a value of 100 means no growth inhibition. A value of 20 would mean 80% growth inhibition. A value of 0 means no net growth over the course of the analysis. A value of −20 would mean 20% lethality. A value of −100 means all cells are dead.

S.3.2. Five-Dose Screen

Candidates that showed remarkable growth inhibition in the one-dose assay were subjected to the 60-cell panel at five concentration levels. The human tumor cell lines of the cancer screening panel were grown in RPMI 1640 medium containing 5% fetal bovine serum and 2 m*M* l-glutamine. Characteristically, cells were injected in 96-well microtiter plates in 0.1 mL at plating densities ranging from 5000 to 40,000 cells/well, depending on the doubling time of individual cell lines. After cell inoculation, the microtiter plates were incubated at 37 °C, 5% carbon dioxide, 95% air, and 100% relative humidity for 24 hours before the addition of tested compounds. After 24 hours, 2 plates of each cell line were fixed *in situ* with Cl_3COOH (TCA), to represent a measurement of the cell population for each cell line at the time of drug addition (t_z). Tested compounds were solubilized in DMSO at 400-fold the desired final maximum test concentration and stored frozen before application. In the course of the drug addition, an aliquot of frozen concentrate was thawed and diluted to twice the desired final maximum test concentration with a complete medium containing 50 μg mL^{-1} gentamicin. Additional four, 10-fold or ½ log serial dilutions were produced to furnish a total of five drug concentrations plus control. Aliquots of 0.1 mL of these different drug dilutions were added to the appropriate microtiter wells already containing 0.1 mL of medium, resulting in the required final drug concentrations.

Following drug addition, the plates were incubated at 37 °C, 5% carbon dioxide, 95% air, and 100% relative humidity for an additional 48 hours. For adherent cells, the test was finished by the addition of cold Cl_3COOH. Cells were fixed *in situ* by the addition of 50 μL of cold 50% (w/v) Cl_3COOH, and incubated at 4 °C for 1 hour. The supernatant was discarded, and the plates were washed with H_2O (5×) and dried in air. Sulforhodamine B (SRB) solution (0.1 mL) at 0.4% (w/v) in 1% CH_3COOH was added to each well, and plates were incubated at RT for 10 min. After staining, the unbound dye was removed by washing five times with 1% CH_3COOH, and the plates were dried in the air. The bound stain is afterward solubilized with a 10 mM trizma base, and the absorbance is read on an automated plate reader at $\lambda = 515$ nm. Utilizing the seven absorbance measurements [time zero (t_z), control growth (c), and test growth in the presence of the drug at the five concentration levels (t_i)], the percentage growth was determined at each of the drug concentration levels. Growth inhibition (%) was calculated as:

$$[(t_i - t_z)/(c - t_z)] \times 100, \text{ for concentrations where } t_i \geq t_z \quad (1)$$

$$[(t_i - t_z)/(t_z)] \times 100, \text{ for concentrations where } t_i < t_z. \quad (2)$$

Three dose–response parameters were calculated as follows. GI_{50} (growth inhibition of 50%) was determined from Equation (3), which is the drug concentration resulting in a 50% reduction in the net protein increase (as measured by SRB staining) in control cells in the course of the drug incubation.

$$[(t_i - t_z)/(c - t_z)] \times 100 = 50 \quad (3)$$
